# Transcription factor Zfp276 drives oligodendroglial differentiation and myelination by switching off the progenitor cell program

**DOI:** 10.1093/nar/gkac042

**Published:** 2022-02-07

**Authors:** Tim Aberle, Sandra Piefke, Simone Hillgärtner, Ernst R Tamm, Michael Wegner, Melanie Küspert

**Affiliations:** Institut für Biochemie, Friedrich-Alexander-Universität Erlangen-Nürnberg, D-91054, Erlangen, Germany; Institut für Biochemie, Friedrich-Alexander-Universität Erlangen-Nürnberg, D-91054, Erlangen, Germany; Institut für Biochemie, Friedrich-Alexander-Universität Erlangen-Nürnberg, D-91054, Erlangen, Germany; Institut für Humananatomie und Embryologie, Universität Regensburg, D-93053, Regensburg, Germany; Institut für Biochemie, Friedrich-Alexander-Universität Erlangen-Nürnberg, D-91054, Erlangen, Germany; Institut für Biochemie, Friedrich-Alexander-Universität Erlangen-Nürnberg, D-91054, Erlangen, Germany

## Abstract

In oligodendrocytes of the vertebrate central nervous system a complex network of transcriptional regulators is required to ensure correct and timely myelination of neuronal axons. Here we identify Zfp276, the only mammalian ZAD-domain containing zinc finger protein, as a transcriptional regulator of oligodendrocyte differentiation and central myelination downstream of Sox10. In the central nervous system, Zfp276 is exclusively expressed in mature oligodendrocytes. Oligodendroglial deletion of Zfp276 led to strongly reduced expression of myelin genes in the early postnatal mouse spinal cord. Retroviral overexpression of Zfp276 in cultured oligodendrocyte precursor cells induced precocious expression of maturation markers and myelin genes, further supporting its role in oligodendroglial differentiation. On the molecular level, Zfp276 directly binds to and represses Sox10-dependent gene regulatory regions of immaturity factors and functionally interacts with the transcriptional repressor Zeb2 to enable fast transition of oligodendrocytes to the myelinating stage.

## Graphical Abstract

**Graphical Abstract:**
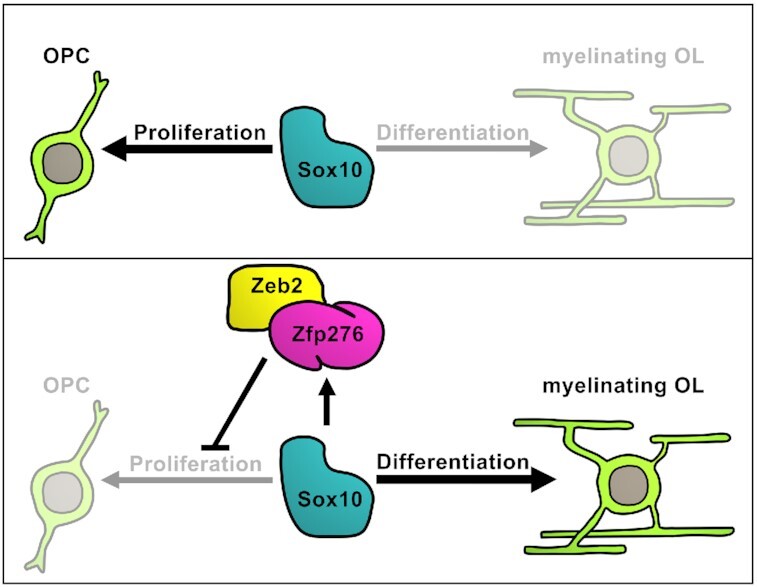
Graphical representation of the proposed pro-differentiating mechanism of Zfp276 in OLs. OPCs can differentiate toward mature OLs or are maintained in the OPC state in the presence of immaturity factors and inhibitors of myelination. Upon differentiation, OLs induce myelination-associated genes to facilitate proper myelination of neuronal axons. Both the genes related to OPC maintenance and those related to terminal differentiation are positively regulated by Sox10. Zfp276, in concert with Zeb2, is able to antagonize the Sox10-dependent activation of OPC-related genes by transcriptional repression of their gene regulatory regions, thereby shifting the scale toward OL differentiation and myelination.

## INTRODUCTION

Ensheathment of neuronal axons by myelinating oligodendrocytes (OLs) is a prerequisite of fast conduction of action potentials in the vertebrate central nervous system (CNS). Oligodendrocyte progenitors (OPCs) are generated from neural precursors of the ventricular zone. Regulation of their proliferation, migration and terminal differentiation relies on the concerted action of several transcriptional regulators, miRNAs and chromatin remodelers (1,2). A key transcriptional regulator of OL development, Sox10, is expressed throughout the oligodendroglial lineage and acts in both, the OPC and OL state, by activation of stage specific gene expression (3,4). To allow proper lineage progression from the OPC to the OL stage, the differentiation program has to be switched on and the proliferation program has to be switched off. Only few transcription factors were identified until now that fulfill the latter task. Therefore, we searched for potential transcription factors that are induced in OLs at the onset of terminal differentiation and are able to switch off the OPC program by direct transcriptional repression of immaturity factors. For initial candidate selection, we screened published RNA expression data sets and ChIP-Seq data sets (5–7) for genes with active chromatin marks and Sox10 binding sites in the CNS as well as enriched mRNA expression in oligodendrocytes. An additional criterion was predicted or verified nuclear localization of the encoded protein according to the UniProt database (http://www.uniprot.org/).

Preliminary qRT PCR and in situ hybridization experiments pointed to the zinc finger protein Zfp276 as a nuclear factor induced in OLs upon terminal differentiation. Zfp276 belongs to the subclass of ZAD (Zinc finger-associated domain) zinc finger proteins and represents the only member of this class in the vertebrate subphylum (8). In contrast to vertebrates, ZAD zinc finger proteins represent the largest group of transcription factors in insects such as Drosophila melanogaster and act as important developmental regulators (9,10).

Using a conditional Zfp276 knockout mouse (Zfp276cko) model, we found that loss of Zfp276 in the OL lineage leads to transiently delayed differentiation and myelination. Forced expression of Zfp276 in primary (1°) rat oligodendroglial cells (OGCs) induced premature expression of maturation markers and myelin genes. We furthermore showed that Zfp276 is a direct target of Sox10 and counteracts the Sox10-dependent activation of regulatory regions of OPC-related genes by directly binding to the regions. Zfp276 therefore antagonizes the activation of immaturity factors by Sox10 during early steps of OL differentiation and therefore shuts off the proliferation program to enable timely OL differentiation and proper onset of myelination.

## MATERIALS AND METHODS

### Transgenic mice

An ES cell clone carrying a Zfp276 knockout ready allele was used to create Zfp276 transgenic mice (Zfp276tm1a, Eucomm Consortium). Crossing these with mice encoding the FLPer recombinase deleted the FRT-flanked neomycin cassette and resulted in a conditional *Zfp276* allele, in which exons 2 to 5 are flanked by loxP-sites (*Zfp276^fl^*). *Sox10::Cre*-mediated (11) recombination of this allele leads to Zfp276 functional deletion in OGCs of the CNS (Zfp276cko). In mice used for 1° mouse OPC culture, an additional *Rosa26^stopfloxtdTomato^*allele (12) was crossed in for identification of Zfp276cko newborn mice. For a CNS-specific Sox10 knockout, *Sox10^fl/fl^* mice (13) were crossed with mice carrying a *Brn4::Cre* allele (14). Genotyping was performed under standard conditions with the primers shown in Supplementary Table S1. Transgenic and control mice were on a mixed C3H x C57Bl/6J background and kept under standard housing conditions with 12 h light–dark cycles and continuous access to food and water, according to animal welfare laws. Both male and female mice were used for analysis.

### Plasmids

For luciferase assays pCMV5-based eukaryotic expression plasmids encoding *Sox10*, *Zeb2/Sip1* and *Zfp276* open reading frames were used. The Sox10 expression vector was described before (15). Expression plasmids for Zfp276 were generated by cloning the coding sequence of either full-length murine Zfp276 (NM_020497.2), or fragments encoding the amino acids 1 to 230 (Nterm), 206 to 433 (Mfrag), or 409 to 614 (Cterm) via BglII/XbaI into pEGFP-C1 (Clontech) or pCMV5. For Zeb2/Sip1 expression plasmids, the Zeb2/Sip1 coding sequence (NM_015753.3) and an N-terminally located 6xmyc tag were inserted into pCMV5 via ClaI/XbaI. For retroviral production, an N-terminally located, myc tag encoding linker region was cloned into the CAG-IRES-EGFP vector via BglII/BamHI sites (16), and the full-length Zfp276 coding sequence was subsequently added in the same reading frame via BglII/BamHI sites. Luciferase reporter constructs with gene regulatory regions from *Wnt7a*, *Id4*, *Tgfb2*, *Cspg4*, *Mag*, *Mbp* and *Plp1* were described before (17–20). The *Zfp276* intronic enhancer and the *Id2* ECR-27kb enhancer were identified using the ECR Browser (https://ecrbrowser.dcode.org/). The luciferase reporter constructs containing the *Zfp276* intronic enhancer (Mm10 chr8:123,257,111–123,257,410) and the *Id2* ECR-27kb (Mm10 chr.12: 25,124,439–25,124,719) were created by PCR amplification of these evolutionary conserved regions from mouse genomic DNA and insertion into the pTATAluc reporter plasmid (21) via KpnI/XhoI or KpnI/NheI sites. The dim-luc luciferase plasmid was previously described (17). Into this plasmid, an optimized W-1 site (W-1 opti) as well as a non-binding W-1 site, where crucial positions 1–3 were mutated (W-1 mut), were introduced downstream of the dimeric Sox10 binding site from the *Mpz* promoter (C-C’) (21) via SacI/KpnI sites. For knockdown experiments, pSuper-Neo-GFP plasmids were used that express Sox10-specific shRNA or scrambled control shRNA (19).

### Cell culture, transfections and viral transduction

N2a neuroblastoma, HEK293T (both from obtained from ATCC, CCL-131 and CRL-1573, respectively) and Oln93 cells (gift of C. Richter-Landsberg) (22) were kept in high glucose DMEM (Gibco, 11965092), supplied with 10%FCS (Gibco, 10500064) and Pen/Strep (Gibco, 15140122). The oligodendroglial CG4 cell line (23) was grown in serum-free SATO (with T3 (Sigma, T6397) and T4 (Sigma, T1775)) (24) or basal medium (DMEM-F12 (Gibco, 11320033) with N2 (Gibco, 17502048), B27 (Gibco, 17504044), 0.01% BSA (Life technologies, 15260) and 100 U/ml Pen/Strep) in the presence of PDGF-AA and bFGF (PeproTech, 100–13A and 100–18B) (25,26), and differentiation was induced by replacement of mitogens with 20 pg/ml CNTF (PeproTech, 450-50).

1° OGCs were obtained from dissected forebrain of either newborn Wistar rats or P0-P3 transgenic and control mice via mixed glial cultures and subsequent shake-off and microglial depletion (27,28). For proliferation, 1° rat OGC cultures seeded on poly-ornithine (Sigma, P3655) coated cell culture dishes were kept in basal medium, supplemented with 10 ng/ml PDGF-AA and 10 ng/ml bFGF. To induce differentiation, medium was changed to SATO medium containing T3, T4 and 20 pg/ml CNTF. 1° mouse OGCs were seeded on poly-d-lysine (Sigma, P6407) and laminin (Sigma, L2020) coated cell culture dishes and kept in the proliferative basal medium supplied with 10 ng/ml PDGF-AA and 10 ng/ml bFGF. Mouse OGCs were differentiated by replacing mitogens with 20 pg/ml CNTF.

Retroviruses were produced by transient transfection of HEK293T cells with Lipofectamin2000 (ThermoFisher, 11668019) and either CAG-myc-IRES-EGFP or CAG-myc-Zfp276-IRES-EGFP plasmids. Retroviruses were enriched by ultracentrifugation and used for transduction. 1° rat OGCs and CG4 cells were transduced at a MOI of 1, and CG4 cells were sorted for EGFP fluorescence prior to ChIP and qRT PCR experiments. After transduction, 1° OGCs were kept in basal medium supplied with bFGF and PDGF-AA for 1 day, and medium was subsequently changed to SATO medium supplied with 20 pg/ml CNTF, in which the cells were kept for up to 6 days with replacement of half the medium on every other day.

For luciferase assays in N2a cells, 0.5 μg of luciferase reporter construct and 0.275 μg pCMV5-based expression plasmids were transfected using 2 μl of Superfect reagent (Qiagen, 301305) according to the manufacturer's instructions in 24-well plates. Overall plasmid amounts were kept constant. The luciferase assay has been described in detail before (29).

### Immunoprecipitation studies

For protein interaction studies, whole cell lysates were produced from transfected HEK293T cells as described (29). Proteins of interest were precipitated with mouse a-myc antibody (Cell Signaling, 9E11), rabbit anti-Zfp276 antiserum (selfmade) or rabbit anti-Sox10 antiserum (selfmade). Precipitation experiments were performed with Protein A-Sepharose beads (G&E Healthcare, 17–0780-01). Proteins were eluted by adding Laemmli sample buffer to a final concentration of 50 mM Tris-HCl, 2% SDS, 1% β-mercaptoethanol, 10% glycerin, 0.1% bromophenol blue and heating for 5 min at 95°C prior to electrophoretic separation on 8–10% SDS-polyacrylamide gels. After western blotting on nitrocellulose membranes, proteins were detected via Luminol (Sigma, A4685) chemiluminescence using the following antibodies: mouse anti-myc (Cell Signaling, 9E11, 1:4000 dilution), rabbit anti-Zfp276 antiserum (self-made, 1:2000 dilution), rabbit anti-Sox10 (self-made, 1:4000 dilution), mouse anti-GFP (Roche, 11814460001, 1:4000 dilution), goat anti-mouse-HRP (KPL, 074–1506, 1:2000 dilution), Protein A-HRP conjugate (Zymed, 1:2000 dilution).

### Immunohistochemistry, immunocytochemistry, *in situ* hybridization and proximity ligation assay

Immunohistochemistry and *in situ* hybridization were performed on 10 μm thick transverse mouse spinal cord sections at forelimb level and coronal mouse brain sections (Bregma −0.5 to +0.5). Mice were killed, and tissue fixed in either 2% (for Zfp276 staining) or 4% paraformaldehyde. Tissue was dehydrated in 30% sucrose and frozen in TissueTek Freezing Medium (Leica). DIG-labeled antisense ribonucleotide probes against *Mbp* and *Plp1* were used for *in situ* hybridization and detected on a Leica MZFLIII stereomicroscope equipped with an Axiocam (Zeiss). For immunohistochemistry, the antibodies and antisera shown in Supplementary Table S2 were used (19,30–32).

For immunocytochemistry, cells were fixed with either 2% or 4% paraformaldehyde. Primary antibodies are listed in Supplementary Table S2. The Zfp276 antibody was generated by immunizing rabbits with a bacterially expressed, purified protein fragment spanning amino acids 260 to 423 of mouse Zfp276 (accession number NP_065243.2). The guinea-pig anti-Myrf antiserum was generated by immunizing guinea-pigs with a bacterially expressed, purified peptide corresponding to amino acids 1 to 184 of mouse Myrf (accession number Q3UR85.2).

For proximity ligation assays, CG4 cells were transfected with pCMV5-myc-Zeb2 using Xfect transfection reagent (Takara Biotech, 631318) according to the manufacturer’s instruction and treated with 2% paraformaldehyde after 3 days under differentiating conditions. The Duolink Proximity Ligation Kit (Sigma-Aldrich, DUO92102) was used according to the manufacturer’s instruction with mouse anti-myc antibody and anti-Zfp276 antiserum (Supplementary Table S2). After the proximity ligation assay, the cells were stained with the anti-myc antibody and anti-Zfp276 antiserum with appropriate donkey-raised Alexa488- and Cy5-coupled secondary antibodies.

Secondary antibodies were coupled to Alexa488 (Molecular Probes, dilution 1:500), Cy3, Cy5 or AMCA (all Dianova, dilution 1:200) raised in donkey. Nuclei were stained with DAPI, unless AMCA-coupled secondary antibodies were used. Sections were analyzed and documented on a Leica DMI 6000B inverted microscope (Leica) equipped with a DFC 360FX camera (Leica).

### Electron microscopy

Spinal cords of control and Zfp276cko mice were dissected at P7 and P30 and transferred to cacodylate-buffered 2.5% paraformaldehyde and 2.5% glutaraldehyde containing fixative. Subsequently tissue was incubated in cacodylate-buffered 1% osmium ferrocyanide, dehydrated, embedded in Epon resin, sectioned transversely, and stained with uranyl acetate and lead citrate. 50 nm sections were examined with a Zeiss Libra electron microscope (Carl Zeiss, Inc.). From electron microscopic pictures, *g*-ratios and number of myelinated axons were determined.

### Electrophoretic mobility shift assay

Electrophoretic mobility shift assays (EMSAs) were performed using ^32^P-labeled double-stranded oligonucleotides (Supplementary Figure S3D) as described (15). An amount of 0.05 μg salmon sperm DNA (Sigma, D1626), sheared to 100–400 bp fragments, was used as an unspecific competitor and the probes were either incubated without cell lysate, with control HEK293T lysates (obtained from cells transfected with EGFP vector or empty pCMV5) or myc-Cterm- or EGFP-Cterm-containing whole cell lysates. For super-shift,1 μl of a rat anti-GFP antibody (Nacalai Tesque, 04404–84, Lot No. M7E5845) was preincubated with cell extracts for 20 min prior to addition of the labelled probes.

### qRT PCR analysis

RNA was harvested from cultured 1° rat or mouse OGCs and CG4 cells (either in undifferentiated state or after 1–6 days in differentiation medium) using the Qiagen RNeasy Micro kit (Qiagen, 74004) and Sox10cko spinal cord using Trizol (Invitrogen, 15596026). After reverse transcription of cDNA with an oligo-dT primer and M-MuLV reverse transcriptase (NEB), quantitative PCR was performed using PowerUp SYBR Green Mastermix (Thermo Fisher Scientific, A25743) with primers shown in Supplementary Table S3. All samples were processed as technical triplicates. Data were analyzed by the ΔΔCt method.

### Chromatin immunoprecipitation

Transduced and EGFP-sorted CG4 cells were cross-linked with 1% paraformaldehyde at room temperature, and chromatin was sheared to fragments of 150–400 bp with a Bioruptor (Diagenode). Subsequently, sheared chromatin was precleared and incubated with either mouse anti-myc (Cell Signaling, 9B11), mouse total IgG, self-made rabbit anti-Zfp276 antiserum, self-made rabbit anti-Sox10 antiserum (31) or the corresponding rabbit pre-immune sera. BSA-blocked Protein A-Sepharose beads (GE Healthcare, 17–0780-01) were used to precipitate chromatin-protein complexes. Crosslinking was reversed via incubation at 65°C for 2 h with proteinase K digestion (Roche, 03115844001), and DNA was purified with the NucleoSpin Gel and PCR cleanup Kit (Macherey-Nagel). Eluted DNA was then used for quantitative PCR, in which the ΔΔCt method was used to calculate the percent recovery of a specific DNA region from the total input. The primers shown in Supplementary Table S4 were used for amplification and detection in q-PCR. Each experiment was performed in three biological replicates.

### RNA-Seq and bioinformatic analysis

Total RNA was isolated from two independent preparations per genotype of three days *in vitro* differentiated (3ddiff) 1° mouse OGC cultures, and each derived from three Zfp276cko mice or three ctrl mice at P2-P4. RNA was isolated using the RNeasy Micro Kit (Qiagen), and RNA samples were treated with DNaseI to remove contaminating DNA. Quality and purity of samples were evaluated using an Agilent 2100 Bioanalyzer (Agilent Technologies Germany). An amount of 100 ng total RNA were used for library preparation (Ilumina TrueSeq Stranded mRNA Kit). Approximately 27 million reads were generated per library using an Illumina Hiseq 2500 platform sequencer (Next Generation Sequencing Core Facility, FAU Erlangen-Nürnberg) and mapped onto mouse genome mm10 using STAR (version 2.5.4.a). Unique mappings were detected using HTSeq count based on ENSEMBL Gene identifier Version 75. Statistical analysis was carried out using DESeq2 R Version 1.18.1. Gene expression values are deposited in GEO under accession number GSE181044. To identify changes in gene expression upon loss of Zfp276 and potential Zfp276 target genes, a list of differentially expressed genes was generated, which displayed a base mean of ≥20, were regulated with a log_2_-fold ≥0.6 and a adjusted *P*-value of ≤0.05. Gene ontology (GO) analysis of differentially regulated genes was performed using the Gene Ontology enrichment, analysis and visualization tool GOrilla (http://cbl-gorilla.cs.technion.ac.il/) in combination with semantic clustering by REViGO for removal of redundant GO terms (http://revigo.irb. hr/) (33). The Gene Set Enrichment Analysis (GSEA) tool from the Broad Institute (http://software.broadinstitute.org/gsea/index.jsp) was used to detect global changes in gene signatures upon loss of Zfp276.

### Quantifications and statistical analysis

Results from independent animals, experiments or separately generated samples were treated as biological replicates. Sample size was *n* ≥ 3 for all mice and molecular biology experiments except for RNA-Seq experiments. Exact sample sizes are given for each experiment. Investigators were not blinded in animal experiments. Fluorescence intensities were quantified using Fiji ImageJ software (W.S. Rasband, U.S. National Institutes of Health, Maryland, USA). GraphPad Prism7 (GraphPad software, La Jolla, CA, USA) was used for statistical testing using two-tailed Student’s *t* tests and analysis of variance (ANOVA) to determine differences in cell or axon numbers, *g*-ratios, luciferase activity, band intensities, transcript level or immunoprecipitated DNA (*P ≤ 0.05; **P ≤  0.01, ***P ≤ 0.001). Variance between statistically compared groups was similar.

## RESULTS

### In the CNS Zfp276 is exclusively expressed in differentiating and myelinating OLs

**Figure 1.**
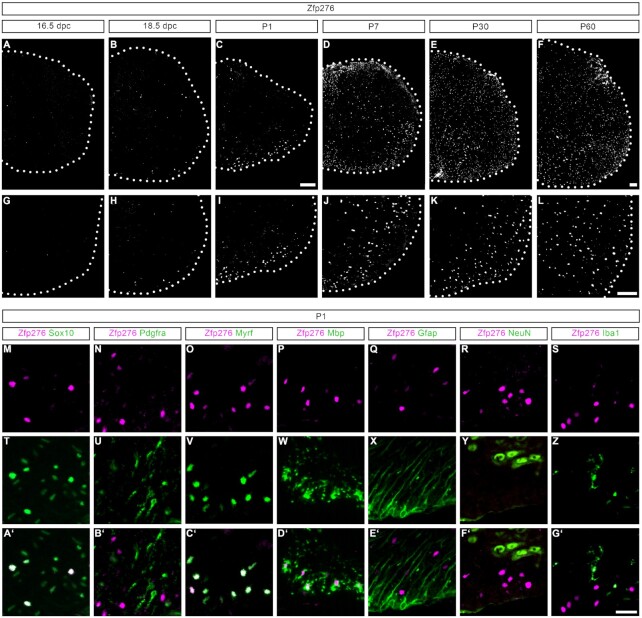
Cell type-specific expression of Zfp276 in differentiating OLs. (**A–L**) Representative immunohistochemical stainings of transverse spinal cord sections from wild-type mice starting at 16.5 days post-coitum (dpc) until postnatal day P60 using anti-sera directed against Zfp276. Panels (A–F) show half a transverse spinal cord section placed on a black background, (G–L) show higher magnification of the ventral white matter; scale bar: 100 μm. (**M–G'**) Representative immunohistochemical stainings of transverse spinal cord sections from wild-type mice at postnatal day 1 (P1) showing co-stainings of Zfp276 (magenta, M–S) with different cell type-specific markers (green, T–Z) and the respective merged channels (overlay white, A’–G’): Sox10 (M, T, A’) Pdgfra (N, U, B’), Myrf (O, V, C’), Mbp (P, W, D’), Gfap (Q, X, E’), NeuN (R, Y, F’) and Iba1 (S, Z, G’); scale bar: 25 μm.

For initial analysis of the Zfp276 expression pattern in the murine CNS, we generated Zfp276-specific antisera directed against amino acids 260–423 of mouse Zfp276 and corresponding to the unique region between the ZAD domain and the pentameric C2H2 zinc fingers. We furthermore focused our analysis on the spinal cord as a model region of the CNS in which oligodendroglial development is regularly studied. Immunohistochemical staining was performed at different embryonic and postnatal ages to detect the cell type- and stage-specific expression pattern of the Zfp276 protein. Interestingly, no staining in any cell type of the spinal cord could be detected before the start of terminal OL differentiation, which takes place at around 18.5 dpc (Figure 1A, G). At this age, the first Zfp276-positive cells were found in the prospective white matter regions (Figure 1B, H). All of them co-expressed the pan-oligodendroglial marker Sox10. In contrast, Zfp276-specific staining was not observed in Gfap-positive astrocytes, NeuN-positive neurons or Iba1-positive microglia (Figure 1M–G′). However, only 11% of all Sox10-expressing OGCs showed staining for Zfp276. Zfp276-positive cells co-expressed the differentiation-associated transcriptional regulator Myrf and the early myelin protein Mbp at P1, but not the OPC marker Pdgfra, identifying them as differentiating OLs (Figure 1N–P, U–W, B′–D′). This was also the case in exemplary white and gray matter regions of the mouse forebrain at P7, when oligodendrocyte development is in a comparable stage as in the perinatal spinal cord. Both in the corpus callosum as well as in the cortex, Zfp276-positive cells overwhelmingly co-express Sox10 (Supplementary Figure S1A, H), the OL maturity marker Myrf and the myelin protein Mbp (Supplementary Figure S1C, D, J, K) but not the OPC marker Pdgfra (Supplementary Figure S1B, I). No cells were found to co-express Zfp276 with the astroglial, neuronal or microglial markers GFAP, NeuN and IbaI (Supplementary Figure S1E–G, L–N). This strongly supports a CNS-wide restriction of Zfp276 to mature myelinating oligodendrocytes.

During the peak period of OL differentiation and spinal cord myelination in the early postnatal spinal cord, the number of Zfp276-positive cells increased steadily to 87% of all oligodendroglial cells at P30 (Figure 1C–E, I–K and Supplementary Figure S1O). The percentage of Zfp276-positive OLs was maintained at high levels in the adult spinal cord (Figure 1F, L and Supplementary Figure S1O). Timing, spatial occurrence and marker colocalization all point to specific functions of Zfp276 during OL differentiation. We additionally performed qRT-PCR analyses on mRNA samples derived from 1° mouse and rat OGC cultures in proliferative and differentiating conditions as well as from the oligodendroglial CG4 cell line. In all three, *Zfp276* transcripts robustly increased upon induction of terminal differentiation and expression remained high at 3 days (3ddiff) and 6 days of differentiation (6ddiff) (Supplementary Figure S1P–R). Immunocytochemical co-staining on 6ddiff rat 1° OGC cultures confirmed that only Mbp-positive mature OLs showed intense signals for Zfp276 (arrows), whereas Mbp-negative OGCs were also negative for Zfp276 (arrowhead) (Supplementary Figure S1S, T). Therefore, 1° OGC cultures derived from early postnatal forebrain tissue recapitulate the induction of Zfp276 upon OL differentiation seen *in vivo* in the mouse spinal cord.

### Forced expression of Zfp276 in 1° rat OGCs induces premature differentiation *in vitro*

**Figure 2.**
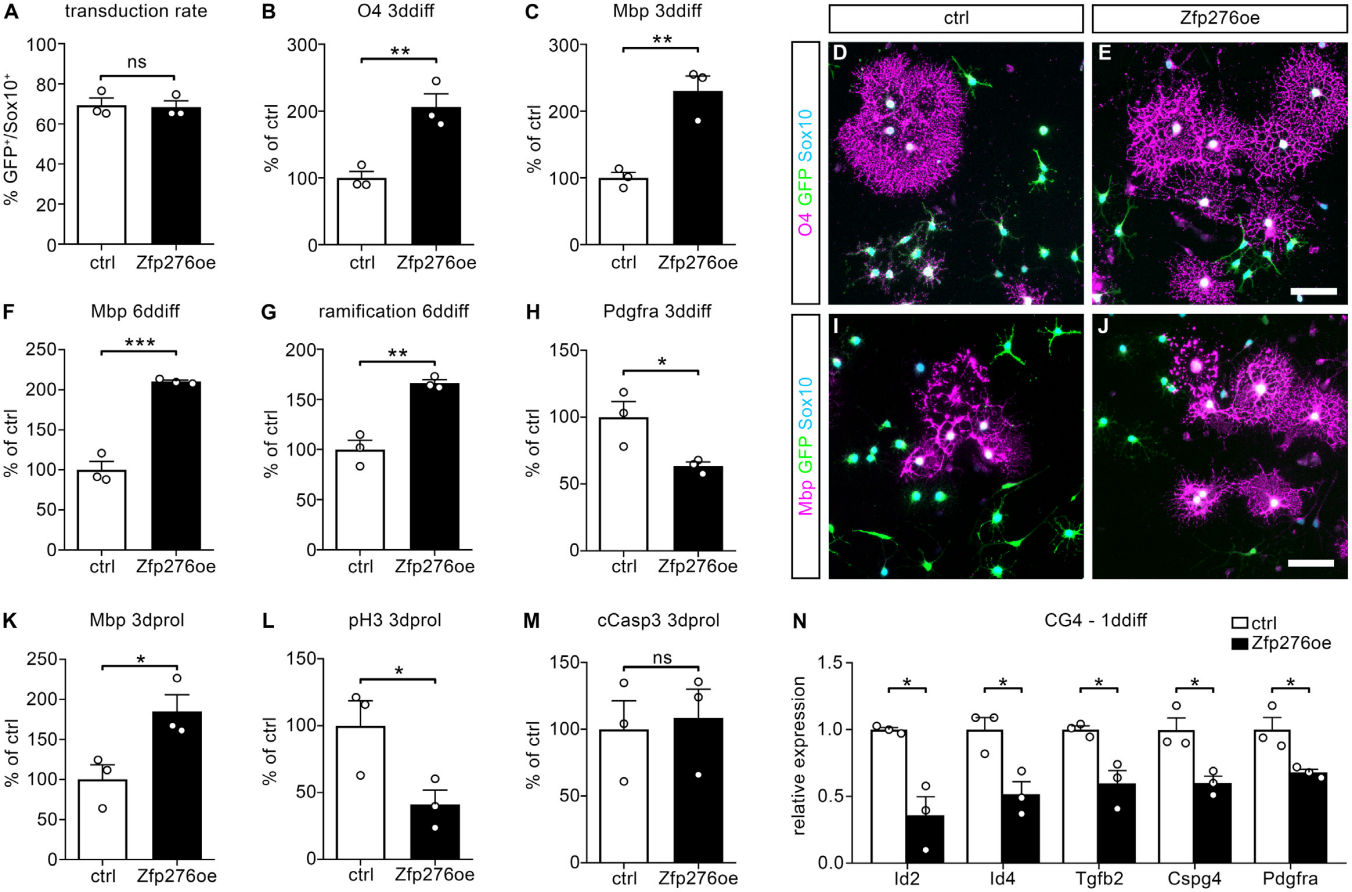
Forced expression of Zfp276 in 1° rat OGCs induces premature differentiation. 1° rat oligodendroglial cells (OGCs) were transduced with an EGFP only virus (ctrl, white bars) or a Zfp276-IRES-EGFP virus (Zfp276oe, black bars). (**A**) Quantification of transduction rates as percent of EGFP/Sox10-double-positive OGCs per total number of Sox10-positive cells (**B, C, F, H**). Quantification of O4- (B), Mbp- (C, F) or Pdgfra- (H) positive oligodendrocytes (OLs) of ctrl or Zfp276oe 1° rat OGCs at 3 or 6 days of differentiation (3ddiff: B-C, H, 6ddiff: F). Number of O4-, Mbp- or Pdgfra-positive transduced OGCs was divided by the total number of Sox10-positive cells and the mean value of the control set to 100% (mean values ± SEM). (**D, E, I, J**) Representative images of 3ddiff (D, E) and 6ddiff (I, J) 1° rat OLs stained for O4 (magenta, D, E) or Mbp (magenta, I, J), EGFP (green) and Sox10 (cyan), transduced with a ctrl (D, I) or Zfp276oe (E, J) retrovirus; scale bar: 50 μm. (**G**) Cell process morphology of transduced OGCs after 6ddiff and stained for Mbp was assessed using an ImageJ software plugin for Sholl analysis and the ramification index of ctrl cells was set to 100% (mean values ± SEM). (**K–M**) Quantification of Mbp- (K), phosphohistone-3 (pH3)- (L) or cleaved Caspase3 (cCasp3)- (M) positive OGCs after 3 days in proliferating conditions (3dprol). Number of marker-positive OGCs were divided by the total number of Sox10-positive cells and the mean value of the control was set to 100% (mean values ± SEM). (**N**) qRT-PCR was performed on total RNA isolated from virally transduced and FACS-purified oligodendroglial ctrl and Zfp276oe CG4 cells using specific oligonucleotides for transcripts of immaturity markers *Id2, Id4, Tgfb2, Cspg4* and *Pdgfra*. Expression levels were normalized to *Gapdh* and *Rpl8*. Ctrl values were arbitrarily set to 1 and relative expression was presented as mean values ± SEM. Statistical analysis was performed with Student’s two-tailed *t*-test (* *P*≤ 0.05, ** *P*≤ 0.01, *** *P*≤ 0.001).

To elucidate Zfp276 functions during OL differentiation, we first analyzed the effects of gain of function of Zfp276 in 1° rat OGC cultures. 1° rat OGCs were transduced with an EGFP only virus (ctrl) or a Zfp276-IRES-EGFP (Zfp276oe) virus. Transduction rates for both viruses were comparable at nearly 70% of all Sox10-positive cells (Figure 2A). Immunocytochemical staining was performed for the early maturation marker O4 at 3ddiff and the mature myelin protein Mbp in combination with Sox10 and EGFP at 3dprol, 3ddiff and 6ddiff. Interestingly, transduction with Zfp276-IRES-EGFP virus led to a 2-fold increase of O4-positive transduced OLs at 3ddiff (ctrl set to 100% ± 10%, Zfp276oe = 188% ± 4%) (Figure 2B, D, E). The number of Mbp-positive transduced OLs was also strongly increased upon forced expression of Zfp276 under conditions that favor OL differentiation (3ddiff: ctrl set to 100% ± 8%, Zfp276oe = 230% ± 22%, 6ddiff: ctrl set to 100% ± 10%, Zfp276oe = 210% ± 2%) (Figure 2C, F, I, J). Forced expression of Zfp276 furthermore led to more elaborate cell processes, visible in a 1.7-fold higher ramification index by Sholl analysis at 6ddiff (ctrl set to 100% ± 9%, Zfp276oe = 166% ± 3%) (Figure 2G). This is indicative of a more differentiated state. The observed increase in differentiating cells was counterbalanced by a decrease in Pdgfra-positive OPCs at 3ddiff (ctrl set to 100% ± 10%, Zfp276oe = 63% ± 2%) (Figure 2H). Interestingly, precocious Zfp276 expression was even able to induce Mbp expression under proliferative conditions (Figure 2K). Concomitantly with the effects on differentiation the number of transduced OGCs expressing phosphohistone H3 as a marker of mitotic cells was significantly reduced upon expression of Zfp276 under proliferating conditions (ctrl set to 100% ± 19%, Zfp276oe = 41% ± 11%) (Figure 2L). No effects were observed on cell survival as judged by cleaved Caspase3-positive OGC numbers at 3dprol (Figure 2M). However, OPC marker expression was substantially reduced in CG4 cells upon overexpression of Zfp276. At 1ddiff, forced Zfp276 expression led to strongly reduced transcript levels of *Id2*, *Id4*, *Tgfb2*, *Cspg4* and *Pdgfra* as OPC maintenance and proliferation genes (36–68% of ctrl, Figure 2N). Our results therefore point to a faster transition into the post-mitotic mature OL stage and to Zfp276 as a positive regulator of OL differentiation.

### Conditional deletion of Zfp276 in the OL lineage leads to a transient delay in OL maturation and myelination

We next analyzed OL lineage progression in the spinal cord of mice carrying an OL-specific Zfp276 deletion. For this purpose, we used a conditional *Zfp276* allele in which exons 2–5 are flanked by loxP sites (*Zfp276^fl^*) (Supplementary Figure S2A). Cre-mediated deletion leads to loss of the ZAD-encoding region of the mRNA and introduction of a frameshift with generation of an early stop codon in exon 6. Therefore, the recombined allele is at most able to generate a protein consisting of the N-terminal amino acids 1–60 and lacking the ZAD domain and all five C2H2 zinc fingers. The conditional *Zfp276^fl^* allele was combined with a *Sox10::Cre* BAC transgene that is active in OGCs from the OPC stage (11), and specific generation of the Zfp276^Δ^ allele only in mice carrying the Cre transgene was verified by allele-specific genotyping PCRs (Supplementary Figure S2B). Comparative staining of spinal cord tissue from Zfp276^fl/fl^ Sox10::Cre mice (Zfp276cko) and Zfp276^fl/fl^ mice (ctrl) with antibodies directed against Zfp276 at P1 argued that a recombination rate of 70% was achieved (Supplementary Figure S2C–E).

**Figure 3.**
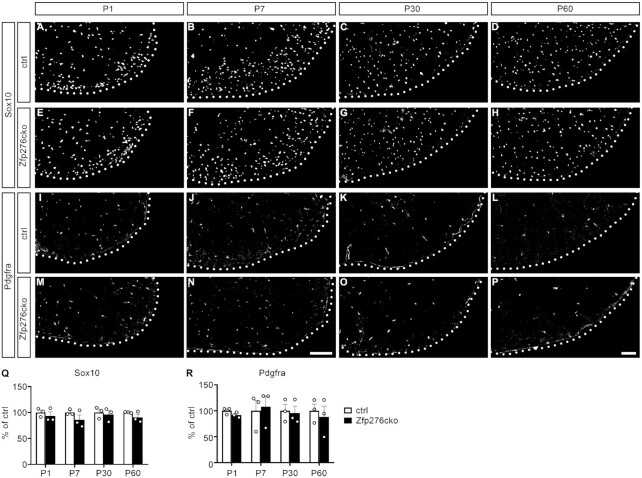
Conditional deletion of Zfp276 in the OL lineage does not change total OGC numbers in the spinal cord. (**A–P**) Representative immunohistochemical stainings of spinal cord sections from control (A–D, I–L) and Zfp276cko (E–H, M–P) mice from P1 to P60 with antibodies directed against Sox10 (A–H) and Pdgfra (I–P). Shown is the right ventral horn, placed on a black background; scale bar: 100 μm. (**Q** and **R**) Quantifications of Sox10- (Q) and Pdgfra- (R) positive OGCs in transverse spinal cord sections of control (white bars) and Zfp276cko (black bars) mice at P1, P7, P30 and P60 (*n* = 3). Number of cells in ctrls was set to 100% (mean values ± SEM). Statistical analysis was performed with Student’s two-tailed *t*-test (* *P*≤ 0.05, ** *P*≤ 0.01, *** *P*≤ 0.001).

To test the function of Zfp276 in the OL lineage *in vivo*, we analyzed OGC marker expression in the spinal cord at different postnatal stages. At all analyzed ages overall OGC numbers were not significantly changed as represented by similar Sox10-positive cell numbers per spinal cord section in control and Zfp276cko mice (Figure 3A–H, Q). Pdgfra-positive OPC numbers were also comparable between control and Zfp276cko mice (Figure 3I–P, R).

**Figure 4.**
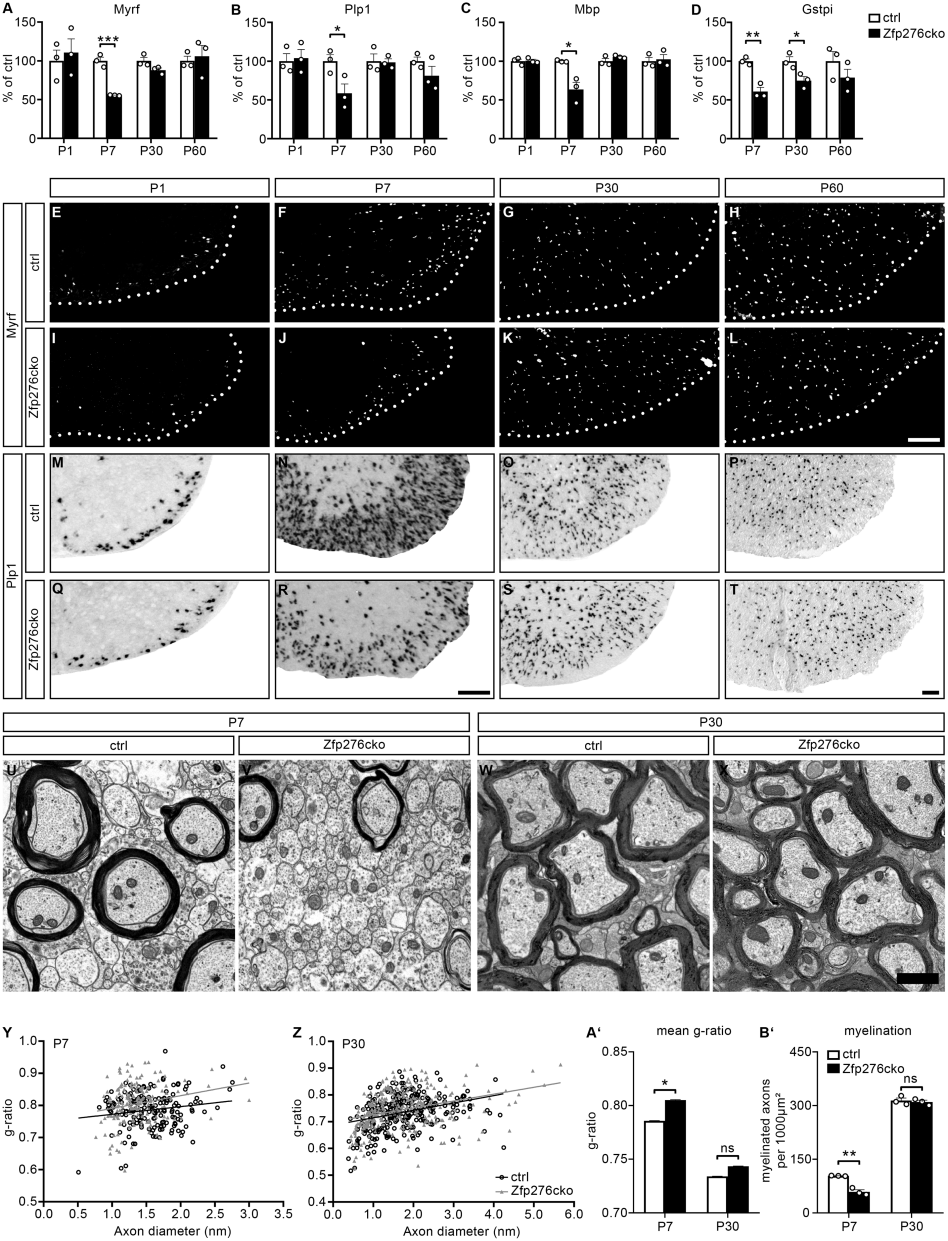
Conditional deletion of Zfp276 in the OL lineage leads to a transient delay in OL maturation and myelination without microglial activation. (**A–D**) Quantification of Myrf (A), Plp1 (B), Mbp (C) and Gstpi (D) expression in transverse spinal cord sections of control (white bars) and Zfp276cko (black bars) mice at P1, P7, P30 and P60. In case of Myrf and Gstpi marker-positive cells were counted, in case of Plp1 at all stages and Mbp at P1 transcript-expressing cells were counted. In case of Mbp from P7 on the area showing Mbp *in situ* signal was measured using ImageJ and divided through total spinal cord area. Mean values of control were set to 100%. Bars indicate mean values ± SEM. (**E–L**) Representative immunohistochemical stainings of spinal cord sections from control (**E**–**H**) or Zfp276cko (**I**–**L**) mice from P1 to P60 with an antiserum directed against Myrf. The right ventral horn placed on a black background is shown; scale bar 100 μm. (**M–T**) Representative *in situ* hybridization of spinal cord sections from control (M–P) or Zfp276cko (Q–T) mice from P1 to P60 using a Plp1 antisense probe. The right ventral horn placed on a white background is displayed; scale bar: 100 μm. (**U–X**) Representative electron microscope pictures from P7 (U, V) and P30 (W, X) control (U, W) and Zfp276cko (V, X) mice. A region from the ventral white matter is shown; scale bar: 1 μm. (**Y** and **Z**) Scatter plot of *g*-ratios for single axons of P7 (Y) or P30 (Z) control (black circles and line) and Zfp276cko (gray triangles and line) spinal cord ventral white matter. At P7, 170 myelinated axons per genotype were measured; at P30, 300 myelinated axons were measured. (**A’**) Mean *g*-ratios of P7 (Y) and P30 (Z) mice were calculated for control (white bars) and Zfp276cko (black bars). SEM were calculated for single axons as separate replicates from two mice per genotype. Data represent mean values ± SEM. (**B’**) Quantification of myelinated axons per 1000 μm² on ultrastructural electron microscope pictures of control (white bars) and Zfp276cko (black bars) mice. Data represent mean values ± SEM. Statistical analysis was performed with Student’s two-tailed *t*-test (* *P*≤ 0.05, ** *P*≤ 0.01, *** *P*≤ 0.001).

We then analyzed the expression of maturity markers in the spinal cord. At P1 no significant changes could be detected for OL numbers. However, a significant reduction to 56% ± 0% of ctrl was detectable for cells expressing Myrf protein at P7, when myelination in the spinal cord is taking place at a maximal rate (Figure 4A, E–L). Cells positive for transcripts of the myelin genes *Mbp* and *Plp1* and Gstpi protein were similarly reduced at this stage in Zfp276cko mice, pointing to an oligodendroglial maturation defect (Figure 4B–D, F, J, N, R, Supplementary Figure S2G, K). At P30 only a slight reduction in Gstpi numbers was still detectable whereas the observed differences in all other differentiation markers and myelin genes were no longer present at P30 and P60 (Figure 4A–D, G–H, K–L, O–P, S–T, Supplementary Figure S2H–I, L–M). This points to a delay in OL differentiation and myelin gene expression upon deletion of Zfp276. Consistent with the OL maturation phenotype EM, analyses of P7 spinal cord white matter regions revealed an increased g-ratio (ctrl = 0.79 ± 0, Zfp27cko = 0.81 ± 0) and a drastically decreased number of myelinated axons in Zfp276cko mice (ctrl = 103 ± 0 myelinated axons per 1000 μm^2^, Zfp276cko = 59 ± 3 myelinated axons per 1000 μm^2^) (Figure 4U, V, Y, A', B'). Again the effect had disappeared at P30 (Figure 4W, X, Z, A', B'). The numbers of Gfap- and Iba1-positive cells were comparable between Zfp276cko and ctrl mice at all analyzed ages (Supplementary Figure S2N–E').

### Altered expression of lipid biosynthesis- and myelin-related genes in Zfp276cko OLs

**Figure 5.**
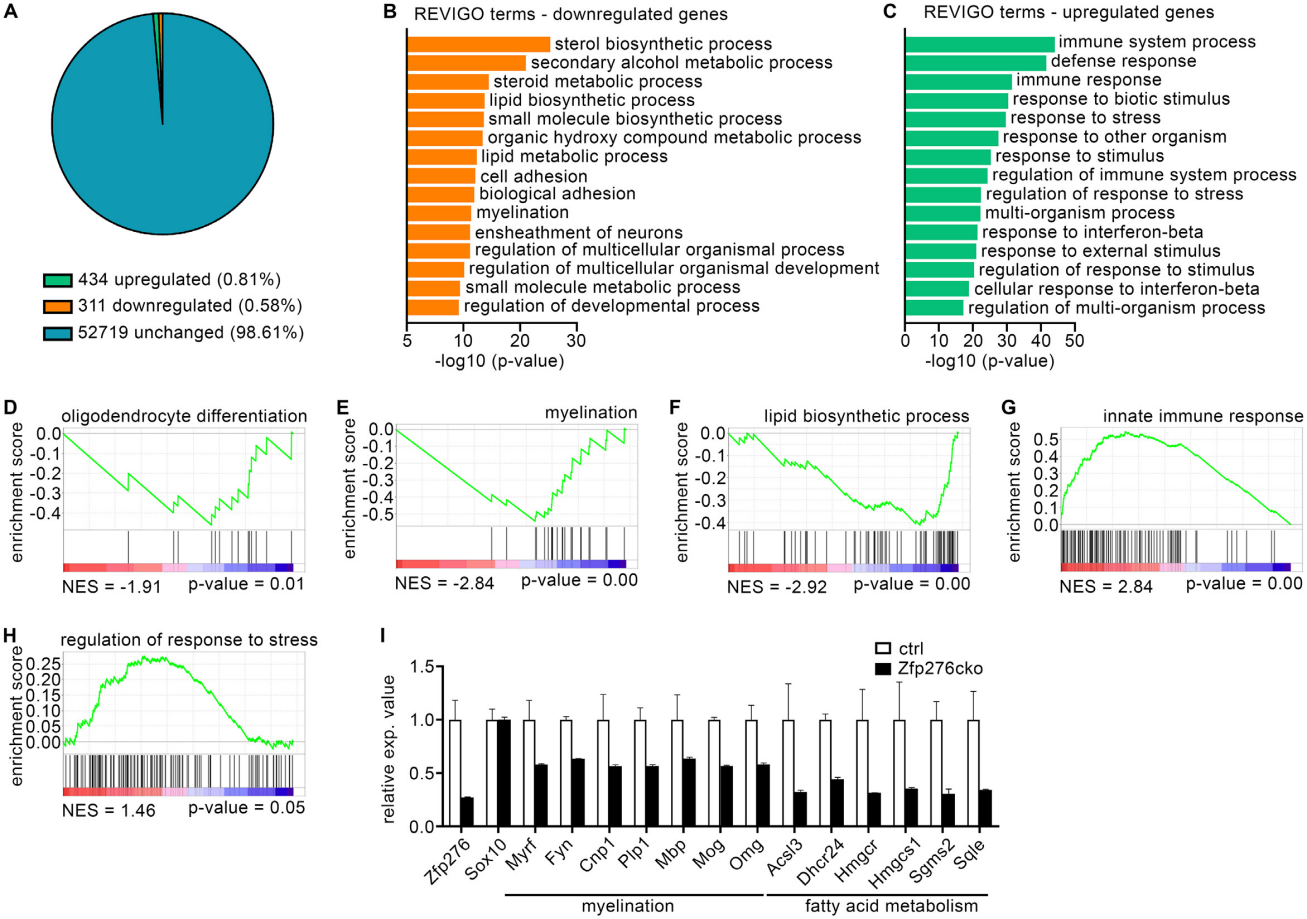
Altered expression of lipid biosynthesis- and myelin-related genes in Zfp276cko OLs. (**A**) Pie chart depicting the percentage of downregulated genes with a log_2_-fold change in expression of ≤ - 0.6 (blue) and upregulated genes with log_2_-fold change in expression of ≥ 0.6 (orange) in Zfp276cko 1° mouse OGCs compared to controls (*P* ≤ 0.05). (**B** and **C**) Gene ontology analysis for biological processes enriched among genes downregulated (B) or upregulated (C) upon loss of Zfp276 in 1° mouse OGCs. Enriched GO terms derived from GOrilla analysis were subsequently reanalyzed using the REVIGO tool and sorted by statistical significance. (**D–H**) Gene set enrichment analysis (GSEA) of regulated genes for enrichment in gene sets related to OL differentiation (D), myelination (E), lipid biosynthetic process (F), innate immune response (G) and regulation of response to stress (H). The normalized enrichment score (NES) is shown in the lower left below each plot, the *P*-value in the lower right. (**I**) Changes in relative expression (exp.) values for Zfp276 and the pan-OGC marker Sox10 as well as for selected genes associated with myelination and fatty acid biosynthesis according to RNASeq data of Zfp276cko (black bars) and control (white bars) OLs.

To identify transcriptomic changes caused by Zfp276 deletion in OLs, we performed RNA-Seq analyses on 1° mouse OGC cultures from ctrl and Zfp276cko mice at 3ddiff *in vitro*. A total of 311 genes were expressed at a lower level in Zfp276cko OLs and 434 genes at a higher level compared to ctrl OLs (log_2_-fold change ≥ 0.6 fold, base mean ≥ 20, *P* ≤ 0.05, Figure 5A). Zfp276 expression was downregulated to 27% ± 0% of ctrl mirroring the incomplete deletion detected by immunohistochemical stainings on postnatal Zfp276cko spinal cord. Sox10 as a pan-oligodendroglial marker was not changed upon Zfp276 deletion as it was also seen *in vivo* in the postnatal spinal cord. Gene ontology (GO) analysis and Gene set enrichment analysis (GSEA) of downregulated genes yielded highest enrichment of genes related to lipid biosynthesis, OL differentiation and myelination (Figure 5B, D–F). Among the strongly downregulated genes in Zfp276cko OLs were regulators of myelination such as Fyn and Myrf, structural components of the myelin sheath such as Cnp1, Plp1, Mbp, Mog and Omg as well as fatty acid, cholesterol and sphingomyelin biosynthetic enzymes such as Acsl3, Hmgcr, Hmgcs1, Dhcr24, Sqle and Sgms2 (Figure 5I). This confirmed the delayed myelination phenotype seen in Zfp276cko spinal cord at P7. Decreased transcript levels of selected downregulated genes were confirmed using separate biological replicates of mouse OGCs in qRT-PCR experiments (Supplementary Figure S2F'). Upregulated GO terms either were rather unspecific terms and correlated to a reactive phenotype of Zfp276-deleted OLs such as response to stimulus or response to stress or were correlated to activation of the immune system (Figure 5C). Similar gene sets were enriched in the GSEA analysis for upregulated genes (Figure 5G–H). Taken together, these data from RNA-Seq on forebrain-derived Zfp276cko OLs confirm the delayed differentiation and hypomyelination phenotype detected in the early postnatal Zfp276cko spinal cord.

### Sox10 directly induces Zfp276 expression by binding to and activating a newly identified *Zfp276* intronic enhancer

**Figure 6.**
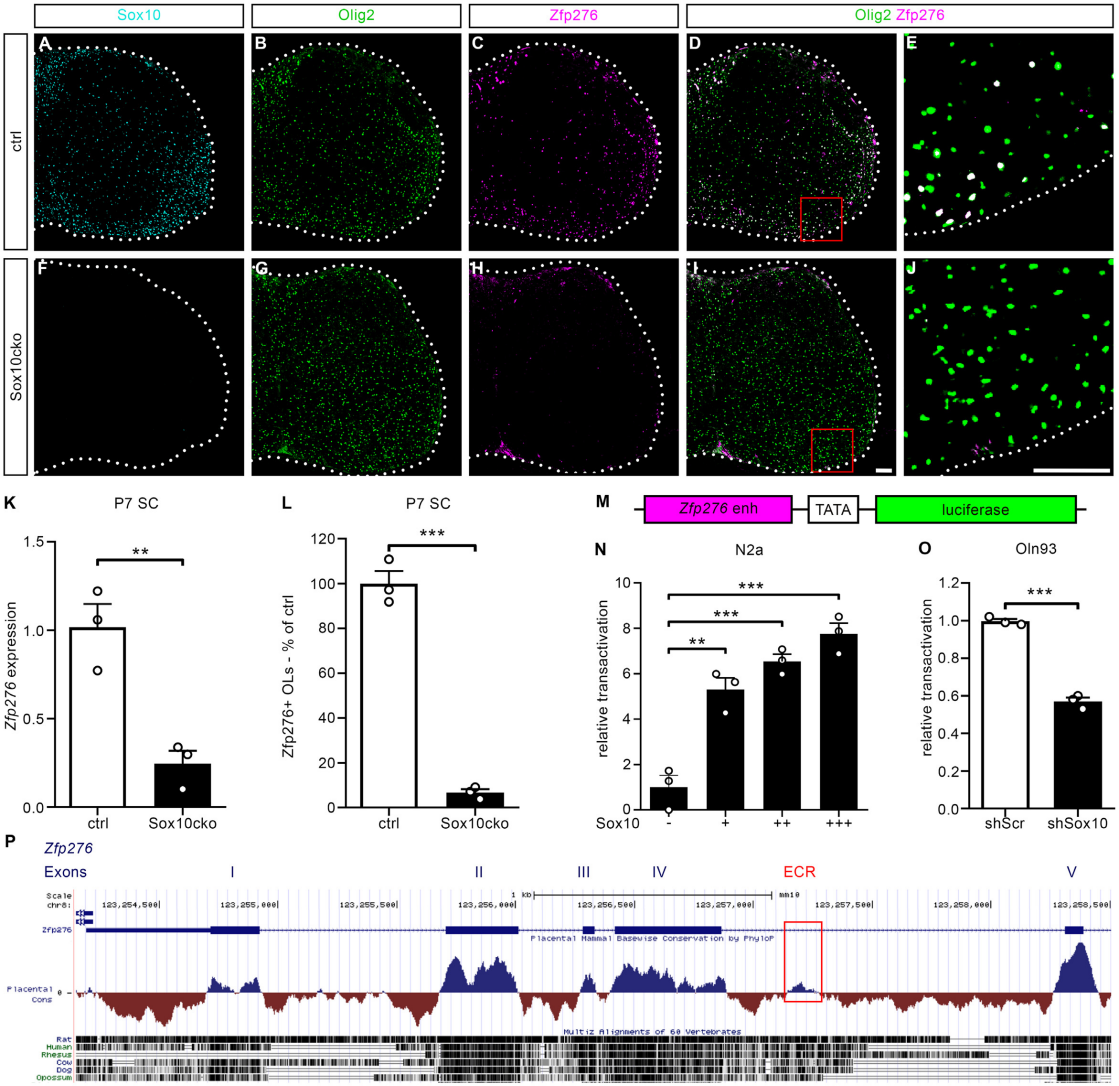
Sox10 directly induces Zfp276 expression by binding to and activating a newly identified *Zfp276* intronic enhancer. (**A–J**) Representative immunohistochemical stainings of the spinal cord from P7 ctrl and Sox10cko mice using antisera against Sox10 (cyan, A, F), Olig2 (green, B, G) and Zfp276 (magenta, C, H) and the respective merged images of Olig2 and Zfp276 signals (overlay white, D, I), and at higher magnification of the ventral white matter (E, J); scale bar: 100 μm. (**K**) *Zfp276* mRNA expression levels in spinal cord extracts from P7 control (ctrl, white bar) or Sox10cko (black bar) mice were determined by quantitative RT-PCR and normalized to *Rpl8* levels. Normalized Zfp276 expression in the ctrl spinal cord was set to 1 and presented as mean values ± SEM. (**L**) Zfp276-positive OGCs from P7 control (ctrl, white bar) or Sox10cko (black bar) mice were counted and the number in ctrl set to 100%. Data indicate mean values ± SEM. (**M**) Schematical representation of the luciferase reporter assay used in (L) and (M). The *Zfp276* ECR (magenta box) and a minimal promoter (TATA, white box) control the expression of a firefly luciferase gene (green box). (**N** and **O**) Transient transfections of the *Zfp276* ECR luciferase reporter construct. (N) In Sox10-negative N2a cells the reporter was co-transfected with either a control plasmid (−) or increasing amounts of Sox10 (+, ++, +++). Reporter gene expression was measured 48 h post-transfection, and effector-dependent activation rates are presented as relative transactivation ± SEM with relative light units of control plasmid-transfected cells arbitrarily set to 1. (O) In Oln93 cells the reporter was co-transfected with either a scrambled shRNA (shScr, white bar) or a Sox10-specific shRNA (shSox10, black bar). Reporter gene expression was measured 72 h post-transfection and presented as relative transactivation ± SEM with relative light units of scrambled shRNA-transfected cells arbitrarily set to 1. (**P**) Localization and evolutionary conservation of the evolutionary conserved region (ECR, red box) within intron 4 of the murine *Zfp276* gene (Generated using the UCSC genome browser; genome configuration: mm10; https://genome.ucsc.edu/). Statistical analysis was performed using Student’s two-tailed *t*-test (* *P*≤ 0.05, ** *P*≤ 0.01, *** *P*≤ 0.001).

To decipher the position of Zfp276 within the OGC transcriptional regulatory network, we analyzed Zfp276 expression in mice in which Sox10 was deleted very early throughout the developing spinal cord by combining the *Sox10^fl^* allele with a *Brn4::Cre* driver (Sox10cko). No Sox10-signal was detectable at P7 in Sox10cko spinal cord sections despite the extensive presence of Olig2-positive OGCs (Figure 6A, B, F, G) (13,14). On transcript level (Figure 6K) as well as on protein level (Figure 6C–E, H–J, L) Zfp276 expression was strongly reduced in P7 spinal cord tissue of these mice. The *Zfp276* mRNA level was reduced to 25% ± 7% of controls. Numbers of Zfp276-positive OLs, identified by Olig2 co-staining, only reached 7% ± 2% of controls. This argues that Zfp276 is genetically downstream of Sox10. To study whether *Zfp276* is a direct target gene, we identified evolutionary conserved regions (ECRs) in the *Zfp276* locus and compared them with Sox10 binding peaks from published ChIP-Seq data sets (6). With this approach we identified a 180 bp region in intron 4 of the rodent *Zfp276* locus (Mm10 chr8:123257179–123257359) and performed reporter gene assays with a construct carrying this ECR in front of a minimal promoter and luciferase open reading frame (Figure 6M–P). Sox10 acts as a dose-dependent activator of this gene regulatory region in the Sox10-negative neuronal cell line N2a (Figure 6N). Compatible with these results, knockdown of Sox10 in the OGC line Oln93 led to reduced activity of this newly identified *Zfp276* enhancer (Figure 6O). Therefore, Sox10 acts as a crucial activator of Zfp276 expression by directly binding to and activating a newly identified intronic *Zfp276* enhancer.

### Zfp276 represses Sox10-dependent gene regulatory regions of OPC proliferation genes and inhibitors of myelination

**Figure 7.**
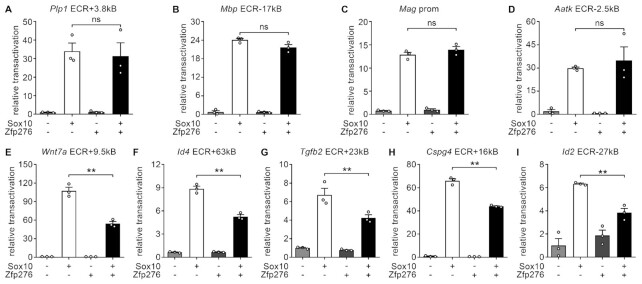
Zfp276 represses Sox10-dependent gene regulatory regions of OPC proliferation genes and inhibitors of myelination. (**A–I**) Luciferase assays in N2a cells with luciferase constructs containing regulatory regions *Plp1* ECR+3.8kb (A), *Mbp* ECR-17kb (B), *Mag* prom (C), *Aatk* ECR-2.5kb (D), *Wnt7a* ECR+9.5kb (E), *Id4* ECR+63kb (F), *Tgfb2* ECR+23kb (G), *Cspg4* ECR+16kb (H) and *Id2* ECR-27kb (I). Co-transfections with empty expression vectors (−) or expression vectors (+) for Sox10 and Zfp276 or a combination of both were performed and mean relative transactivation ± SEM is depicted with empty expression vector set to 1. Statistical analysis: Student’s two-tailed *t*-test (* *P*≤ 0.05, ** *P*≤ 0.01, *** *P*≤ 0.001).

To better define the transcriptional activity of Zfp276, we analyzed its behavior in reporter gene assays on oligodendroglial regulatory regions that had previously been shown to be localized in open chromatin in OGCs, to be bound by oligodendroglial transcription factors *in vivo* and to be activated by Sox10 in reporter gene assays. These included regions from genes related to OPC proliferation and maintenance, such as *Wnt7a*, *Id2, Id4, Tgfb2*, *Cspg4* as well as from genes related to OL differentiation and myelination, such as *Plp1, Mbp*, *Mag* and the *miR338* host gene *Aatk* (17,34). The evolutionary conserved regulatory region 27kb upstream of the mouse *Id2* gene (*Id2 ECR-27kb*) was newly identified in this study as a further Sox10-inducible region. In our experiments, we wanted to test if Zfp276 is able to act as activator or repressor on its own or if it is able to modulate Sox10-dependent activation of any of those gene regulatory regions. In contrast to other transcriptional regulators of OL differentiation, expression of Zfp276 alone could not induce reporter constructs containing regulatory regions of OL differentiation genes (Figure 7A–D). Zfp276 was also unable to synergistically activate the reporters with Sox10. Therefore, Zfp276 probably does not act as direct inducer of maturation markers and myelin genes. However, co-expression of Zfp276 with Sox10 was able to robustly repress Sox10-dependent activation of OPC-related gene regulatory regions (Figure 7E–I). Induction rates were substantially lowered for all five analyzed OPC-specific regulatory regions to as compared to Sox10 alone (from 107 ± 6 to 54 ± 3 for *Wnt7a* [Figure 7E], from 9 ± 0 to 5 ± 0 for *Id4* [Figure 7F], from 7 ± 1 to 4 ± 0 for *Tgfb2* [Figure 7G], from 66 ± 2 to 44 ± 1 for *Cspg4* [Figure 7H] and from 6 ± 0 to 4 ± 0 for *Id2* [Figure 7I]).

Reduced activation of OPC-related gene regulatory regions could be caused by sequestration of Sox10 from its binding sites. To test if Zfp276 and Sox10 proteins interact, we performed co-immunoprecipitation (CoIP) experiments using extracts from HEK293T cells transfected with Sox10 and Zfp276 expression vectors. We were unable to detect a physical interaction between Zfp276 and Sox10 independent of whether we used Zfp276- or Sox10-specific antisera for precipitation (Supplementary Figure S3A, B).

### Zfp276 binds to specific Zfp276 binding sites within gene regulatory regions of its target genes

**Figure 8.**
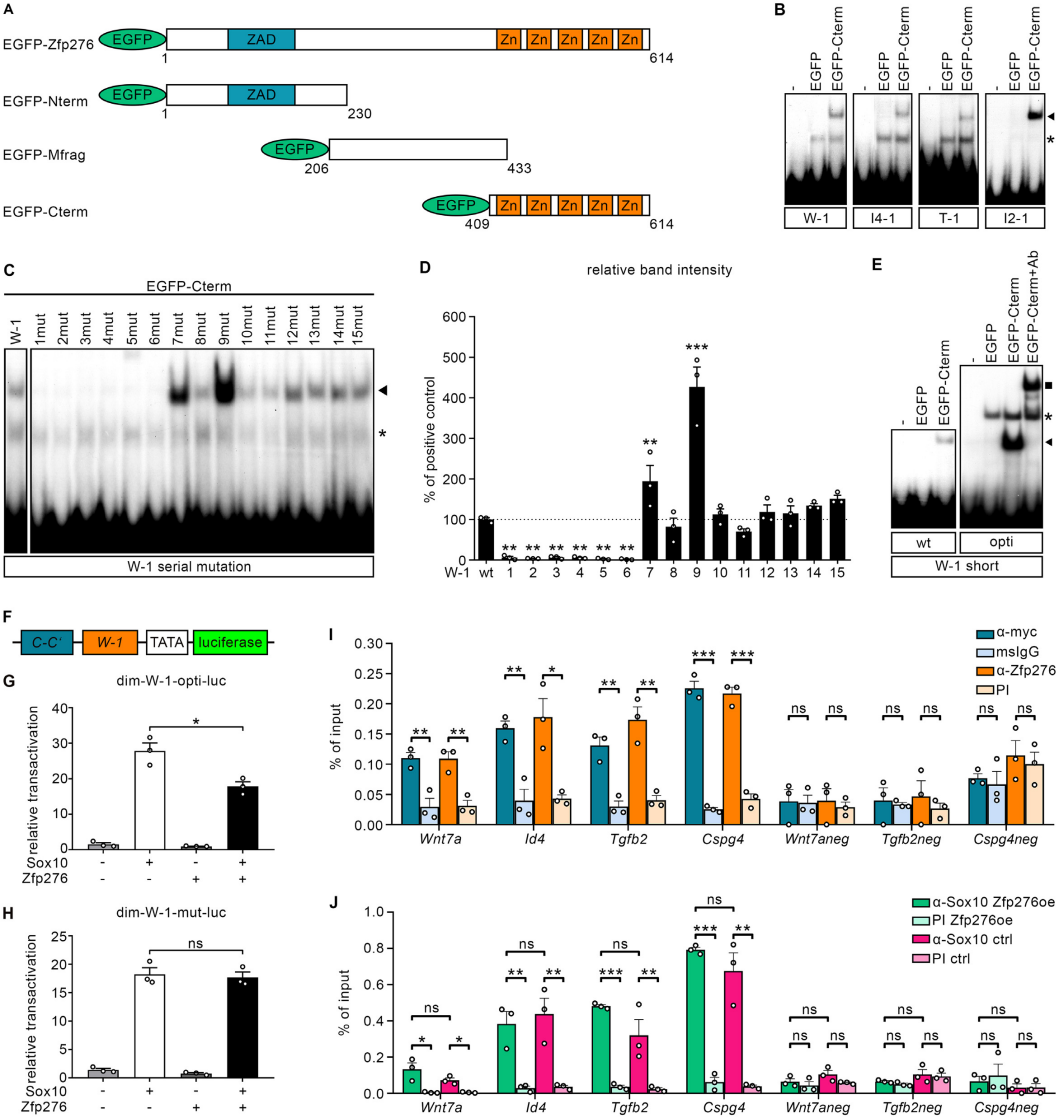
Zfp276 binds to Sox10-dependent gene regulatory regions of OPC proliferation genes and inhibitors of myelination to fulfil its repressive function. (**A**) EGFP-tagged Zfp276 constructs: Full-length Zfp276 (aa1-614), with the zinc-finger associated domain (ZAD) and a pentameric zinc (Zn)-finger domain, was split in three fragments: N-terminal (EGFP-Nterm), middle (EGFP-Mfrag) and C-terminal fragment (EGFP-Cterm). (**B**−**E**) EMSAs using probes with predicted Zfp276 sites in the regulatory regions of *Wnt7a* (W-1), *Id4* (Id4-1),*Tgfb2* (T-1) and *Id2* (Id2-1) (B) (sequences in Supplementary Figure S3D) without cell extracts (−) or with extracts from HEK293T cells expressing EGFP or EGFP-Cterm. Specific bands are marked by an arrowhead, unspecific bands by an asterisk, supershift by a square. (C) EMSA of a mutation series of the native W-1 site. (D) Densitometric analysis of (C). Band intensities were normalized to the native W-1 site and depicted as mean ± SEM. Statistical analysis: One-way ANOVA with Dunnett's multiple comparison test comparing to W1 (* *P*≤ 0.05, ** *P*≤ 0.01, *** *P*≤ 0.001). (E) EMSA of shortened W-1 (wt) and optimized W-1 site (opti). (**F**−**H**) Luciferase assays in N2a cells with the dim-W-1-luc reporter construct shown in (F) carrying a dimeric Sox10 site (C-C’), a minimal promoter (TATA) and the optimized or mutated W-1 site (G-H). Co-transfections with empty expression vectors (−) or expression vectors (+) for Sox10 and Zfp276 or a combination of both were performed and mean relative transactivation ± SEM is depicted with empty expression vector set to 1. Statistical analysis: Student’s two-tailed *t*-test (* *P*≤ 0.05, ** *P*≤ 0.01, *** *P*≤ 0.001). (**I** and **J**) ChIP experiments on ctrl or myc-Zfp276-expressing (Zfp276oe) CG4 cells to detect enriched binding of myc-Zfp276 (I) or endogenous Sox10 (J) to regulatory regions or negative control regions of OPC maintenance genes. Used antibodies: Mouse anti-myc (blue), mouse IgG (light blue), rabbit anti-Zfp276 (orange), rabbit pre-immune serum (PI, light orange), rabbit anti-Sox10 (Zfp276oe: green, ctrl: red) or rabbit pre-immune serum (Zfp276oe: light green, ctrl: pink) (*n* = 3). Amounts of precipitated chromatin were determined for the regulatory regions of the *Wnt7a, Cspg4, Tgfb2* and *Id4* genes and for the related negative control regions (*Wnt7aneg, Tgfb2neg, Cspg4neg*) by qPCR and mean percent of input was displayed ± SEM. Statistical analysis: Student’s two-tailed *t*-test (* *P*≤ 0.05, ** *P*≤ 0.01, *** *P*≤ 0.001).

Next we wanted to test, whether Zfp276 directly binds to the regulatory regions of OPC genes that it influences in their activity. Since the sequence requirements of Zfp276 for DNA binding were not known, we employed an online prediction tool (35,36). For Zfp276, 15 bp binding sequences were predicted and ranked by probability using both a linear and a polynomial algorithm. The Zfp276-responsive gene regulatory regions were then screened for similarities to the predicted Zfp276 binding sites, and the two hits with the highest score per regulatory region were further analyzed in electrophoretic mobility shift assays (EMSAs). A fusion protein between EGFP and the C-terminal 5 zinc fingers of Zfp276 (EGFP-Cterm, Figure 8A) bound to at least one site within the gene regulatory regions of *Id4* (Id4-1) and *Tgfb2* (T-1), *Wnt7a* (W-1) and *Id2* (Id2-1) (Figure 8B). Similar results were obtained with a fusion protein between a myc tag and the C-terminal zinc finger region of Zfp276 (Supplementary Figure S3C).

We used the W-1 site within the *Wnt7a* enhancer to characterize Zfp276 binding properties even further and mutated each nucleotide from positions 1 to 15 to the non-complementary purine or pyrimidine base (Figure 8C, D and Supplementary Figure S3D). Mutations at positions 1 to 6 led to strong reduction of Zfp276 binding (Figure 8D). Mutations at positions 7 and 9 on the other hand increased binding affinity (Figure 8C, D). This led us to define a 9 bp core binding sequence for Zfp276 (5′-AAAAGGGCT-3′). Omission of the flanking positions did not decrease binding affinities, while changes at positions 7 and 9 drastically increased it (W-1 opti: 5′-AAAAGGTCG-3′). The shortened and optimized W-1 binding site (W-1 short opti) carrying both affinity enhancing mutations in positions 7 and 9 showed drastically increased interaction with the Zfp276 C-terminus in EMSA compared to the corresponding wildtype sequence (W-1 short wt) (Figure 8E). Addition of GFP-specific antibodies supershifted the protein−DNA complex and confirmed the presence of Zfp276 in the complex (Figure 8E).

To test the functionality we inserted the optimized Zfp276 binding site (W-1 opti) as well as a non-binding control site (W-1 mut) in an artificial Sox10-responsive reporter gene construct (dim-luc) (Figure 8F). Reporter assays showed that both constructs were robustly activated by Sox10. However, the presence of Zfp276 elicited a decrease in Sox10-dependent activation (from 28 ± 2-fold to 18 ± 1-fold) that was only observed in the presence of the optimized Zfp276 binding site but not its mutant version (Figure 8G, H). To test if this sequence-specific binding is recapitulated in living cells, we performed ChIP experiments on chromatin of oligodendroglial CG4 cells that were transduced with a myc-Zfp276 encoding retrovirus. Chromatin was harvested after 3ddiff and immunopreciptitation was performed using either monoclonal antibody against the myc tag or selfmade Zfp276-specific antisera. For each of the 4 analyzed OPC gene regulatory regions 3- to 9-fold enrichment was detected from chromatin for both antibodies compared to controls (Figure 8I). In contrast, no significant enrichment was found for ctrl genomic regions without Zfp276 consensus motif in the vicinity of the *Wnt7a*, *Tgfb2* and *Cspg4* loci (*Wnt7aneg, Tgfb2neg, Cspg4neg*). Despite forced Zfp276 expression, all OPC regulatory regions could also be immunoprecipitated with antibodies against Sox10 (Figure 8J). Those results argue that Zfp276 specifically binds to the analyzed ECRs in the context of living oligodendroglial cells and fulfills its function as a repressor of Sox10-dependent activation likely without displacing Sox10 from these regions.

### Zfp276 directly interacts with the transcriptional repressor Zeb2/Sip1

**Figure 9.**
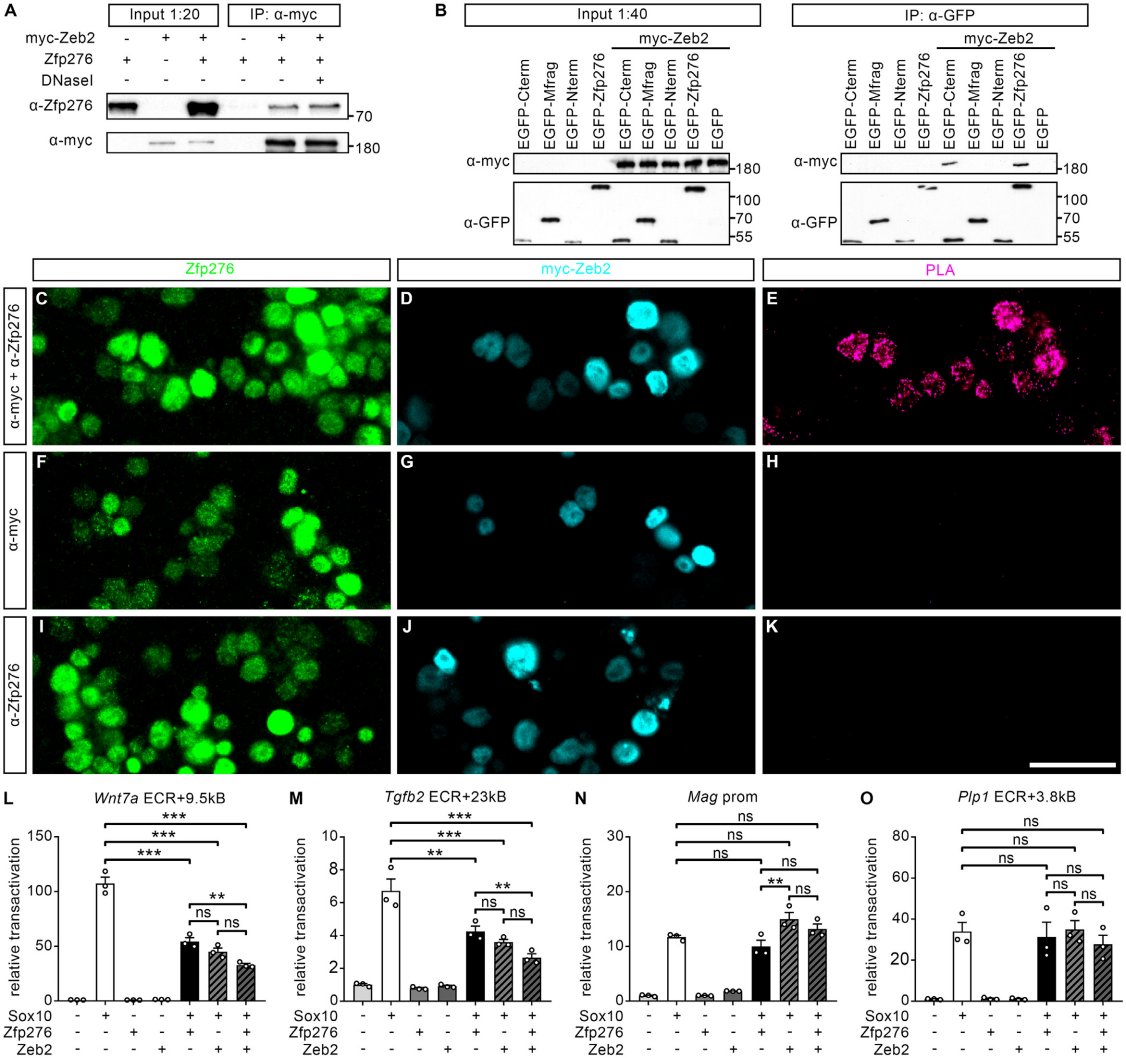
Zfp276 directly interacts with the transcriptional repressor Zeb2 to repress common target genes. (**A**) CoIPs with HEK293T lysates overexpressing either myc-Zeb2, Zfp276 or a combination in the presence or absence of DNaseI. Precipitations were performed with anti-myc antibody. Precipitated myc-Zeb2 was detected with an anti-myc antibody and co-precipitated Zfp276 was detected with antiserum directed against Zfp276. Numbers on the right indicate the position of co-electrophoresed size markers in kDa. (**B**) CoIPs of myc-Zeb2 and EGFP-tagged Zfp276 fragments. HEK293T lysates containing myc-Zeb2 were co-incubated with EGFP, EGFP-Zfp276, EGFP-Nterm, EGFP-Mfrag or EGFP-Cterm lysates together with anti-GFP antibody. Precipitated EGFP-Zfp276 and EGFP-Zfp276 fragments were detected using anti-GFP antibody, co-immunoprecipitated myc-Zeb2 with anti-myc antibody. Numbers on the right indicate the position of co-electrophoresed size markers in kDa. (**C–K**) Proximity ligation assays (PLAs) were performed on CG4 cells transfected with myc-Zeb2 expression vector prior to 3ddiff and subsequent immunocytochemical staining was performed for endogenous Zfp276 (green) and myc-Zeb2 (cyan). Fixed CG4 cells were incubated with either both anti-myc and anti-Zfp276 antibodies (C–E) or either primary antibody alone (F–K) as controls. (**l–O**) Luciferase assays in N2a cells transiently transfected with luciferase reporter vectors containing the same regulatory regions of *Wnt7a* ECR+9.5 kb (L), *Tgfb2* ECR+23 kb (M), *Mag* prom (N) and *Plp1* ECR+3.8kb (O) as described before. Reporter plasmids were co-transfected with empty expression vectors (−) or expression vectors (+) for Sox10, Zfp276 and Zeb2 or combinations as indicated. Reporter gene expression was determined 48 h post-transfection and relative transactivation rates are presented ± SEM. Transfections with empty expression vector only were arbitrarily set to 1. Statistical analysis was performed with one-way ANOVA with Tukey’s multiple comparison test (ns *P* > 0.05, * *P*≤ 0.05, ** *P*≤ 0.01, *** *P*≤ 0.001).

Zeb2/Sip1 is a transcription factor that has been reported to promote OL differentiation by repression of OPC genes (37). So far, Zeb2/Sip1 has been shown to interfere with Bmp and Wnt/beta-catenin signaling and thereby repress inhibitors of differentiation. To test whether Zeb2/Sip1 may additionally affect Sox10 activity and do so in cooperation with Zfp276 we tested if Zfp276 interacts with Zeb2 and performed CoIP experiments using cell extracts of Zfp276- and myc-Zeb2-overexpressing HEK293T cells. Indeed, we were able to precipitate Zfp276 with myc-specific antibodies when both proteins were present and independent of the presence of DNaseI (Figure 9A). Additionally, we wanted to narrow down the interaction domain of Zfp276 with Zeb2. Therefore we performed CoIP experiments with myc-Zeb2 and various Zfp276 regions (Figures 8A and 9B). We found binding of myc-Zeb2 to the C-terminal fragment of Zfp276, where the C2H2 zinc finger domains are located. To verify interaction of Zeb2 and Zfp276 in living cells, we transfected oligodendroglial CG4 cells with myc-Zeb2, let the cells differentiate for 3 days to induce endogenous Zfp276 expression and performed proximity ligation assays (PLAs) (Figure 9C−K). A specific PLA signal was detected only in cells that were treated with both specific antibodies for myc-Zeb2 and endogenous Zfp276, but not with the single antibodies (Figure 9E, H, K). To evaluate the functional relevance of this interaction we repeated the luciferase reporter gene assays on gene regulatory regions of OPC- and OL-related genes, this time in the presence of Zfp276, Sox10 and Zeb2 (Figure 9L−O). Similar to Zfp276, Zeb2 by itself had a repressive effect on Sox10 activation of the *Wnt7a* and *Tgfb2* ECRs but not on that of the *Mag* promoter or the *Plp1* ECR. In the presence of both effectors an even stronger additive repressive effect was detectable for the OPC-related genes (Figure 9L−M).

Taken together, those findings point to a direct physical and functional interaction of Zfp276 and Zeb2 proteins in OLs.

## DISCUSSION

For the very first time, we present a function for the only ZAD zinc finger protein in mammals by showing that Zfp276 acts as a direct regulator of OL differentiation and CNS myelination. Its expression within the spinal cord is restricted to the OL lineage where it starts with the onset of terminal differentiation. Using a conditional Zfp276 knockout mouse model, we detected a transient delay in the expression of OL differentiation markers and myelination of axons in early postnatal stages, revealing a role of Zfp276 as a relevant factor for timely OL differentiation. These findings were supported by RNA-Seq data from ctrl and Zfp276cko OLs and verified by overexpression studies in 1° rat OGC cultures.

Using a DNA binding site prediction tool for zinc finger proteins (35), we identified Zfp276 binding sites within gene regulatory regions of genes involved in maintenance of OPC identity, proliferation and migration or repression of OL differentiation, such as *Cspg4*, *Tgfb2*, *Id2*, *Id4* and *Wnt7a* (34,37–43). Most of these regions are already described to be bound by Sox10 and activated by this transcription factor during the OPC stage (17,18). Expression of these genes has to be shut down in order to allow efficient transition into the myelinating OL stage. Zfp276 seems to do just that and exert its pro-differentiation function through direct binding and repression of the before mentioned regulatory regions of genes that are activated by Sox10 in OPCs. In support, endogenous expression of OPC genes is strongly reduced upon forced expression of Zfp276 in rat OGCs and CG4 cells and the Sox10-dependent activity of the associated regulatory regions is repressed by Zfp276 in luciferase reporter gene assays (Figure 7E–I). Zfp276 therefore is a new transcriptional regulator of oligodendroglial development that acts by repression of progenitor state determinants. In this study, we also characterized the exact DNA-binding preferences of Zfp276 on naturally occurring sites and on the sequentially mutated W-1 site. Comparison of all identified sites led to the Zfp276 consensus (5′-RAAAGGGYK-3′, R = A or G, Y = T or C and K = T or G). Nucleotide identities at the outermost positions are more variable than the core identities and one mismatch at positions 7 to 9 is compatible with binding (Supplementary Figure S3D). Screening of published Sox10-responsive ECRs and promoters of OL differentiation genes including the analyzed regions of the *Aatk/miR338*, *Mag, Mbp* and *Plp1* genes complemented by the *Myrf* enhancer ECR9, the *Cx47* promoter 1b and the *Cx32* promoter (19,44,45) did not identify any Zfp276 consensus sites. In contrast, all the analyzed regions of OPC maintenance and proliferation genes harbor experimentally validated Zfp276 binding sites that are highly conserved and completely match the consensus, or in case of the *Cspg4* intronic enhancer carry a single mismatch in an outer position. This suggests a requirement of direct Zfp276 binding to its target regions to fulfil its regulatory function as repressor of differentiation inhibitors and is an important step in identifying Zfp276 targets on a global scale. Genome-wide screening for the consensus binding site as well as ChIP-Seq experiments may identify further targets of Zfp276 during OL development.

Like Zfp276, the transcription factor Zeb2 influences glial differentiation and myelination not by activating the differentiation program but by terminating the progenitor cell program (37,46). In the CNS, Zeb2 was found to interact with Smad proteins, antagonize the Bmp and Wnt signaling pathways and thereby interfere with expression and function of various inhibitors of OL differentiation that are prevalent in OPCs, such as Hes1, Id2 and Id4 (37).

As Zeb2 and Zfp276 both function by repressing genes essential for the OPC state, we analyzed whether both proteins functionally interact. Here, we show that Zeb2 in addition to its previously determined functions also inhibits the Sox10-dependent activation of *Wnt7a* and *Tgfb2* gene regulatory regions and does so in cooperation with Zfp276 (Figure 9L–M). The joint effect is presumably mediated through physical interaction of the C-terminus of Zfp276 with Zeb2. The combinatorial action of both proteins could be important to effectively terminate the OPC state and thereby facilitate the transition to OL differentiation and upregulation of myelin genes during developmental myelination. In other tissues including the PNS, Zeb2 function was shown to depend on interaction with co-repressors such as CtBP1/2 or the NuRD complex (47–49). Therefore future experiments may tackle the question if Zfp276 function similarly depends on corepressor interactions. In Drosophila, where a massive expansion of ZAD zinc finger proteins from a single ancestor gene took place, several ZAD proteins are described to have architectural functions and support or block long-distance enhancer-promoter interactions (50,51). This may represent an additional mechanism for Zfp276 functions that deserves future analysis as well.

By identification and functional analysis of an intronic evolutionary conserved enhancer within the Zfp276 gene, we furthermore identified Zfp276 as a direct target of Sox10. In previous studies, Sox10 was shown to induce expression of factors that in turn modulate its own function during development of myelinating glial cells. This phenomenon was found for Oct6 and Egr2 in Schwann cells of the PNS and for Myrf in OLs. In most cases this interaction was a positive one, leading to synergistic activation of Sox10 targets (19,52–55). Instead Zfp276 appears to modulate Sox10 function by repressing its activity on target genes. The modulatory role of Oct6, Egr2 and Myrf furthermore involves direct interaction with Sox10. In case of Zfp276, however, no such direct interaction with Sox10 was detected. Sox10 enrichment at its target loci was also not changed upon overexpression of Zfp276 (Figure 8J). It thus seems that Zfp276 appears to inhibit Sox10 activity without direct physical contact.

While our results provide clear evidence that Zfp276 is downstream of Sox10, they cannot fully explain why Zfp276 is selectively expressed in OL and not in OPCs where Sox10 is equally present. Similar observations have previously been made for Oct6 and Myrf. It is therefore likely that additional factors have a critical influence on Zfp276 expression. To identify such additional regulators, we tested known transcriptional activators and inducers of OL differentiation, such as Nfatc2, Myrf and Znf24 for their ability to activate the newly identified *Zfp276* intronic enhancer. However, none increased the activity of the *Zfp276* enhancer alone or in synergism with Sox10 (data not shown). Interestingly, *Zfp276* transcript levels are only moderately upregulated during OL differentiation compared to Zfp276 protein levels (Figure 1A–L). This points to the relevance of post-transcriptional regulation of Zfp276 expression via stage-specific miRNAs. Using published miRNA expression data sets in combination with the miRNA target prediction program TargetScan (http://www.targetscan.org), we identified the OPC-enriched microRNAs *miR-145, miR-214* and *miR-497* as potentials Zfp276 regulators with binding potential to the 3′-UTR of *Zfp276* mRNA. Two of these microRNAs, *miR-145* and *miR-214*, have already been implicated in oligodendroglial differentiation by other studies (25,56–58). These miRNAs need to be experimentally validated regarding their impact on Zfp276 levels in oligodendroglial cells in future studies.

In demyelinating diseases such as multiple sclerosis, efficient remyelination is frequently not achieved because of a blockade of OPC maturation. Such a blockade may be caused by an inefficient exit from the OPC stage. Because of its relevance for leaving the OPC stage, Zfp276 may also influence the remyelination process. Zfp276 may even have a stronger effect on remyelination than on developmental myelination as previously observed for other transcriptional regulators of OL differentiation such as Klf9 and Olig1 (59–62). Future studies will have to tell whether this is the case.

## DATA AVAILABILITY

All data generated or analyzed during this study are included in this published article and its supplementary file or have been deposited in the GEO database (accession number GSE181044).

## Supplementary Material

gkac042_Supplemental_File
