# Supplementary material for: Transcription factor Zfp276 drives oligodendroglial differentiation and myelination by switching off the progenitor cell program
Source: Nucleic Acids Res. 2022 Feb 7;50(4):1951–68. doi: 10.1093/nar/gkac042 (PMC8887482; doi:10.1093/nar/gkac042)
Supplement: gkac042_Supplemental_File [file gkac042_supplemental_file.pdf]

Supplementary figure S1

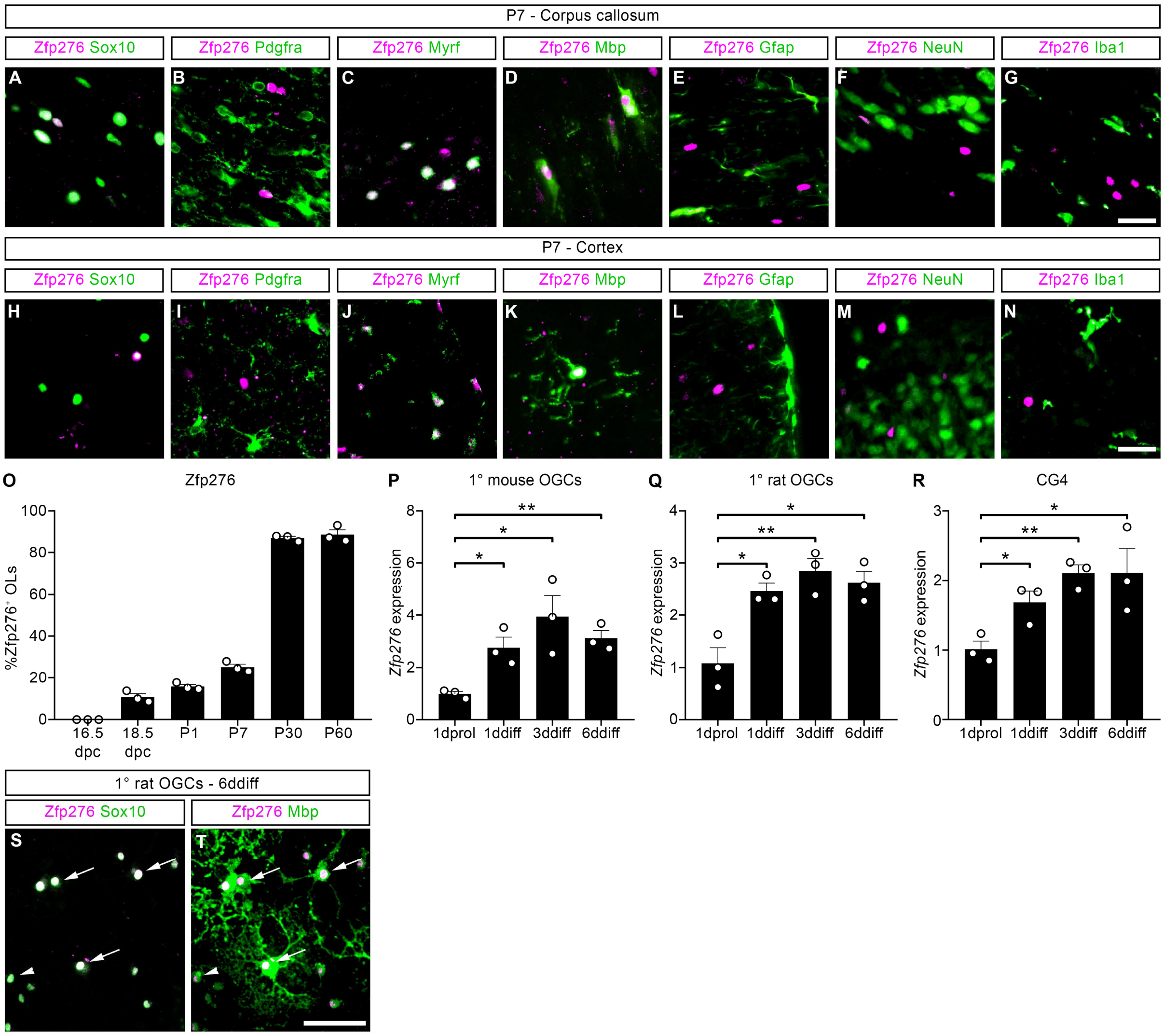

Supplementary figure S1: Expression study for Zfp276 in OGCs

(A-G, H-N) Representative immunohistochemical stainings of coronal sections of corpus callosum (A-G) and cortex (H-N) of wild-type mice at postnatal day 7 (P7) showing co-stainings of Zfp276 (magenta) with different cell type-specific markers (green): Sox10 (A, H) Pdgfra (B, I), Myrf (C, J), Mbp (D, K), Gfap (E, L), NeuN (F, M) and Iba1 (G, N). Scale bar: 25 µm. **(O)** Quantification of Zfp276-positive OLs in transverse spinal cord sections from wild-type mice aged 16.5 dpc to P60 as shown in Figure 1 (A-L). **(P-R)** Determination of *Zfp276* mRNA expression levels by quantitative RT-PCR in 1° mouse OGCs (P), 1° rat OGCs (Q) and the oligodendroglial CG4 cell line (R) under either proliferative (1dprol) or differentiating conditions for 1, 3 or 6 days (1ddiff, 3ddiff and 6ddiff). Expression levels were normalized to *Gapdh* and *Rpl8*. Ctrl values were arbitrarily set to 1 and relative expression was presented as mean values ± SEM. Statistical analysis was performed with Student's two-tailed t-test (* p≤0.05, ** p≤0.01, *** p≤0.001). **(S, T)** Immunocytochemical staining of 6ddiff 1° rat OGCs for Zfp276 (magenta), Sox10 (S, green) or Mbp (T, green). Arrows indicate cells that are positive for Zfp276, Mbp and Sox10; arrowheads indicate a cell that is only positive for Sox10. Scale bar: 50 µm.

Supplementary figure S2

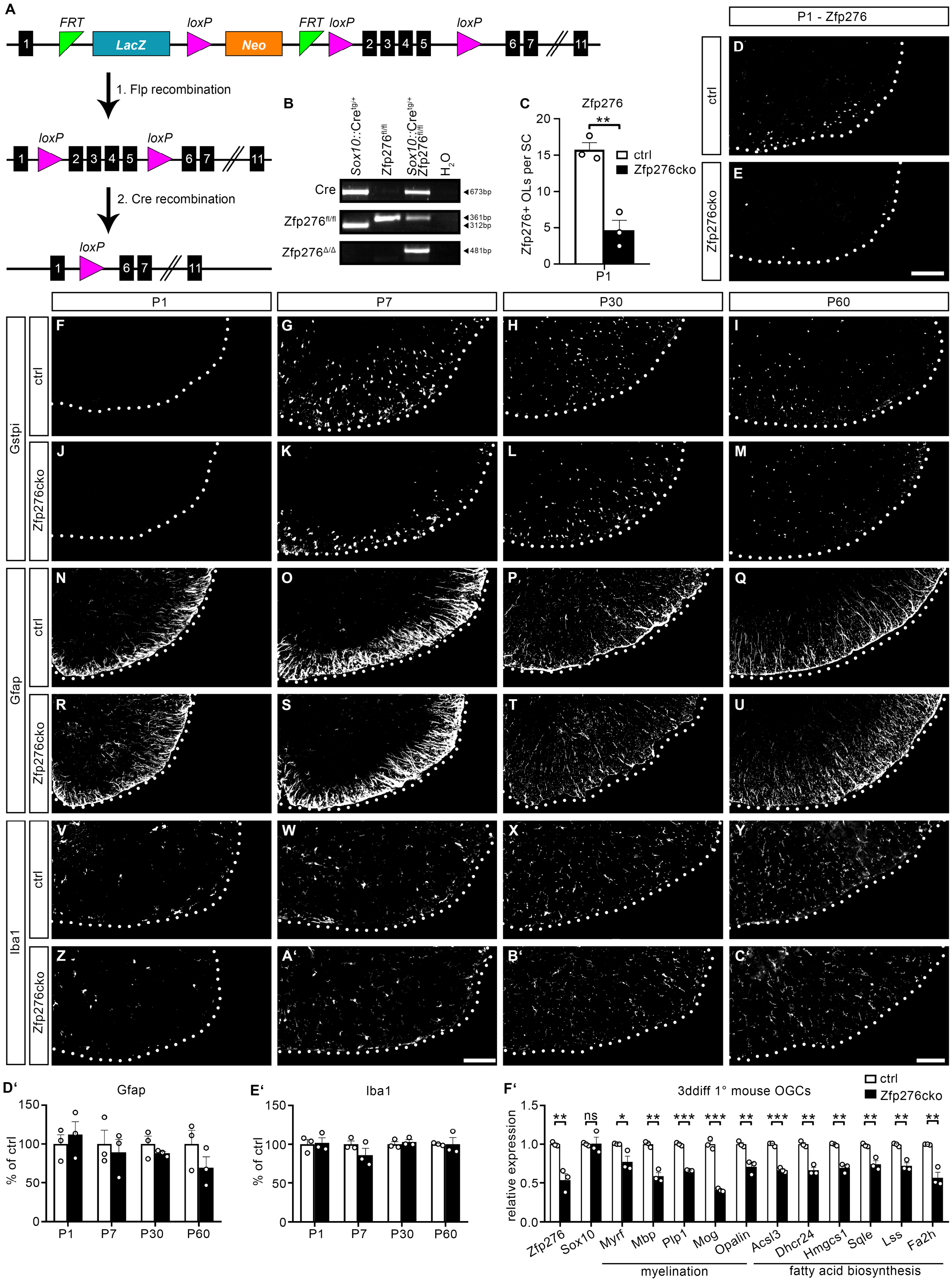

Supplementary figure S2: Conditional deletion of Zfp276 in the oligodendroglial lineage

(A) Schematic representation of the EUCOMM *Zfp276* allele that was used to create the conditional knockout of Zfp276 (Zfp276cko). The allele contains a lacZ-Neo cassette flanked by FRT sites, as well as loxP sites upstream of exon 2 and downstream of exon 5 of the murine *Zfp276* gene. Mating of this mouse line with a Flp recombinase-transgenic mouse line leads to excision of the lacZ-Neo cassette from the allele. Mating of the resulting mouse line with *Sox10::Cre*-transgenic mice results in the

conditional deletion of exons 2 to 5 in the oligodendroglial lineage. **(B)** Representative genotyping PCR specific for the *Cre* transgene, the non-recombined *Zfp276^{fl}* allele (upper band) or the *Zfp276^{wt}* allele (lower band) and the recombined *Zfp276$^{\Delta}$* allele for the genotypes (*Sox10::Cre$^{tg/wt}$, Zfp276$^{wt/wt}$*), (*Sox10::Cre$^{wt/wt}$, Zfp276$^{fl/fl}$*) and (*Sox10::Cre$^{tg/wt}$, Zfp276$^{fl/fl}$*). **(C)** Quantification of Zfp276-positive OGCs in transverse spinal cord sections from ctrl (white bars) and Zfp276cko (black bars) mice at P1. **(D, E)** Representative immunohistochemical stainings at postnatal day P1 of ctrl and Zfp276cko mice stained with a Zfp276 antiserum. **(F-C')** Representative immunohistochemical stainings of P1 to P60 control (F-I, N-Q, V-Y) and Zfp276cko (J-M, R-U, Z-C') transverse spinal cord sections with antibodies against Gstpi (F-M), Gfap (N-U), and Iba1 (V-C') The ventral white matter is shown, placed on a black background. Scale bar 100 µm. **(D', E')** Quantifications of immunohistochemical stainings of transverse spinal cord sections of control (white bars) and Zfp276cko (black bars) mice at P1 to P60 stained with antibodies against Gfap (D') and Iba1 (E'). For Gfap, the corrected total fluorescence was measured with ImageJ and the value of controls set to 100%. For Iba1, Iba1-positive cells were counted, and the numbers of the control set to 100%. Data represents mean ± SEM. **(F')** Determination of transcript levels of selected candidate genes by quantitative RT-PCR in 1° mouse OGC cDNA of ctrl and Zfp276cko mice, which were differentiated for 3 days. Expression levels were normalized to *Gapdh* and *Rpl8*. Ctrl values were arbitrarily set to 1 and relative expression was presented as mean values ± SEM. Statistical analysis was performed with Student's two-tailed t-test (* p≤0.05, ** p≤0.01, *** p≤0.001).

Supplementary figure S3

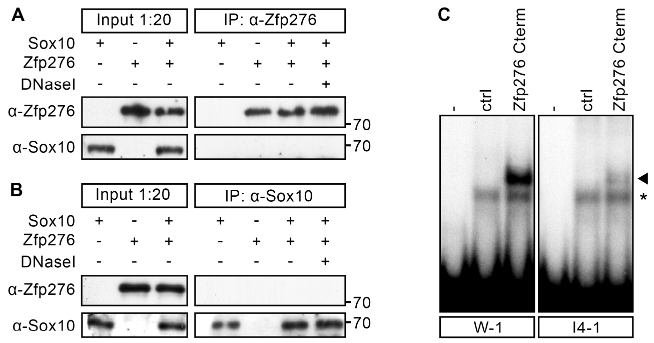

D

										1	2	3	4	5	6	7	8	9	10	11	12	13	14	15										
Id4 (I4-1)	A	A	A	A	A	A	A	G	T	A	A	A	A	G	G	G	C	T	T	T	T	G	C	T	G	A	A	A	G	C	C	T		
Id2 (I2-1)		A	A	T	G	G	G	G	T	G	A	A	A	G	G	G	T	G	G	A	T	T	T	G										
Tgfb2 (T-1)	C	T	C	T	G	G	C	T	T	G	A	A	A	G	G	G	C	T	G	C	T	G	C	T	G	T	G	T	G	A	T	T		
Wnt7a (W-1)			G	G	T	A	A	A	G	A	A	A	A	G	G	G	C	T	T	T	T	G	C	T	T	G	G	G	C	A	A	G	A	T
W-1 serial mutation 1mut			G	G	T	A	A	A	G	C	A	A	A	G	G	G	C	T	T	T	T	G	C	T	T	G	G	G	C	A	A	G	A	T
W-1 serial mutation 2mut			G	G	T	A	A	A	G	A	C	A	A	G	G	G	C	T	T	T	T	G	C	T	T	G	G	G	C	A	A	G	A	T
W-1 serial mutation 3mut			G	G	T	A	A	A	G	A	A	C	A	G	G	G	C	T	T	T	T	G	C	T	T	G	G	G	C	A	A	G	A	T
W-1 serial mutation 4mut			G	G	T	A	A	A	G	A	A	A	C	G	G	G	C	T	T	T	T	G	C	T	T	G	G	G	C	A	A	G	A	T
W-1 serial mutation 5mut			G	G	T	A	A	A	G	A	A	A	A	T	G	G	C	T	T	T	T	G	C	T	T	G	G	G	C	A	A	G	A	T
W-1 serial mutation 6mut			G	G	T	A	A	A	G	A	A	A	A	G	T	G	C	T	T	T	T	G	C	T	T	G	G	G	C	A	A	G	A	T
W-1 serial mutation 7mut			G	G	T	A	A	A	G	A	A	A	A	G	G	T	C	T	T	T	T	G	C	T	T	G	G	G	C	A	A	G	A	T
W-1 serial mutation 8mut			G	G	T	A	A	A	G	A	A	A	A	G	G	G	A	T	T	T	T	G	C	T	T	G	G	G	C	A	A	G	A	T
W-1 serial mutation 9mut			G	G	T	A	A	A	G	A	A	A	A	G	G	G	C	G	T	T	T	G	C	T	T	G	G	G	C	A	A	G	A	T
W-1 serial mutation 10mut			G	G	T	A	A	A	G	A	A	A	A	G	G	G	C	T	G	T	T	G	C	T	T	G	G	G	C	A	A	G	A	T
W-1 serial mutation 11mut			G	G	T	A	A	A	G	A	A	A	A	G	G	G	C	T	T	G	T	G	C	T	T	G	G	G	C	A	A	G	A	T
W-1 serial mutation 12mut			G	G	T	A	A	A	G	A	A	A	A	G	G	G	C	T	T	T	G	G	C	T	T	G	G	G	C	A	A	G	A	T
W-1 serial mutation 13mut			G	G	T	A	A	A	G	A	A	A	A	G	G	G	C	T	T	T	T	T	C	T	T	G	G	G	C	A	A	G	A	T
W-1 serial mutation 14mut			G	G	T	A	A	A	G	A	A	A	A	G	G	G	C	T	T	T	T	G	A	T	T	G	G	G	C	A	A	G	A	T
W-1 serial mutation 15mut			G	G	T	A	A	A	G	A	A	A	A	G	G	G	C	T	T	T	T	G	C	G	T	G	G	G	C	A	A	G	A	T
W-1 opti			G	G	T	A	A	A	G	A	A	A	A	G	G	T	C	G	T	T	T	G	C	T	T									
Zfp276 consensus										**R**	**A**	**A**	**A**	**G**	**G**	**G**	**Y**	**K**																

Supplementary figure S3: Zfp276 binds to specific Zfp276 binding sites within gene regulatory regions of its target genes

(A, B) CoIP experiments were performed with either an anti-Zfp276 (A) or anti-Sox10 (B) antibody using HEK293T lysates containing Zfp276, Sox10 or a combination in the presence or absence of DNaseI. The corresponding antisera were used to detect precipitated Sox10 and Zfp276. Numbers on the right indicated the position of co-electrophoresed size markers in kDa. **(C)** EMSAs using myc-tagged Zfp276 C-terminal fragment (myc-Cterm). Radiolabelled oligonucleotides containing the W-1 and I4-1 site were incubated either without cell lysate, with control lysates or with lysates containing myc-Cterm. Specific shifts are marked by an arrowhead, unspecific bands by an asterisk. **(D)** Oligonucleotide sequences used in EMSAs. Mutated bases are highlighted in red. The putative 15bp Zfp276 binding sequence is marked in gray. The derived Zfp276 binding consensus is depicted in bold type.

Locus	Primers
Cre transgene	5'-ATGCTGTTTCACTGGTTATG-3'
	5'-ATTGCCCCTGTTTCACTATC-3'
Rosa26^{stopfloxtdTomato}	5'-AAGGGAGCTGCAGTGGAGTA-3'
	5'-CCGAAAATCTGTGGGAAGTC-3'
	5'-CTGTTCCTGTACGGCATGG-3'
	5'-GGCATTAAAGCAGCGTATCC-3'
Zfp276^{fl} allele	5'-GGCAACTGGGTGAGACGCAG-3'
	5'-GCAATTCGGACTGCTGTGGG-3'
Zfp276$^{\Delta}$ allele	5'-GGTTAACAGCCTCCATGTGCA-3'
	5'-GCAATTCGGACTGCTGTGGG-3'

Supplementary table 1: Oligonucleotides used for genotyping.

Antibody/-serum	Producer	Lot No.	Cat. No.	Dilution IHC	Dilution WB/ChIP	Dilution ICC/PLA	Citation
rabbit anti-Zfp276	self-made			1:500	1:2000/ 1:125	1:1000	
guinea pig anti-Sox10	self-made			1:500			(30)
rabbit anti-Sox10	self-made				1:4000/1: 125		(31)
goat anti-Sox10	self-made			1:500			(32)
guinea pig anti-Myrf	self-made			1:500			
rabbit anti-Myrf	self-made			1:500			(19)
rabbit anti-cleaved Caspase3	Cell Signaling Technology	0043	9661			1:200	
mouse anti-Gfap	Chemicon		MAB3402	1:100			
chicken anti-GFP	Aves Labs	GFP879484	GFP-1020			1:2000	
mouse anti-GFP	Roche		11814460001		1:4000		
rat anti-GFP	Nacalai Tesque	M7E5845	04404-84			1:4000	
rabbit anti-Gstpi	ENZO Life Sciences	8081910	ADI-MSA-101-E	1:2000			
rabbit anti-Iba1	WAKO	CAP4688	019-19741	1:200			
rabbit anti-Ki67	NeoMarkers	9106S906D	RM-9106	1:500			
rat anti-Mbp	Serotec	120111	MCA409S	1:500		1:500	
mouse anti-myc	Cell Signaling Technology		2276S		1:4000	1:1000	
mouse anti-NeuN	Chemicon	19040027	MAB377	1:500			
mouse anti-O4	R&D systems	HWW1115081	MAB1326			1:500	
rabbit anti-Olig2	Millipore	2060464	AB9610	1:1000			
goat anti-Pdgfra	R&D Systems	HMQ0218081	AF1062	1:50			
rabbit anti-Pdgfra	Santa Cruz	E-1210	sc-338			1:1000	
rabbit anti-phosphohistone3	Upstate Biotech.	19046	06-570			1:1000	

Antibody/-serum	Producer	Lot No.	Cat. No.	Dilution IHC	Dilution WB/ChIP	Dilution ICC/PLA	Citation
goat anti-mouse-HRP	KPL	130535	074-1506		1:2000		
Protein A-HRP conjugate	Zymed		101023		1:2000		

Supplementary table 2: Antibodies used for immunohistochemistry (IHC) and -cytochemistry (ICC), western blot (WB), Chromatin immunoprecipitation (ChIP) and Proximity ligation assay (PLA).

Transcript	Primers
Zfp276	5'-GAGCTGCCCACCATCTACAAG-3'
	5'-GATGAGCTTCACGTGTCGCTG-3'
Id2	5'-CCCGGTGGACGACCCGATG-3'
	5'-CAGATGCCTGCAAGGACAGGATGC-3'
Id4	5'-CTGCGTTGGCCAGGTGT-3'
	5'-AATTTCTGCTCTGGCCCTCC-3'
Tgfb2	5'-AACACACCAAAGTCCTCAGC-3'
	5'-TGGTCAGTGGTTCCAGATCC-3'
Cspg4	5'-GCATGAGATATCCTCTGAGCC-3'
	5'-GAAGAGTACATCATGCCGACC-3'
Pdgfra	5'-CCTCCTTCTACCACCTCAGC-3'
	5'-GGAACAGGGTCAATGTCTGG-3'
Sox10	5'-ACAGCAGCAGGAAGGCTTCT-3'
	5'-TGTCCTCAGTGCGTCCTTAG-3'
Myrf	5'-CTGGAGACTCGCATTGATGA-3'
	5'-GGGACCACTGGTGATGACTT-3'
Mbp	5'-GCTTCAGACCATCCAAGAAGACC-3'
	5'-GGACTACTGGGTTTTCATCTTGG-3'
Plp1	5'-CAAACACCAGGAGCCATACAACA-3'
	5'-GGATTGTGTTTCTTTGGAGTGG-3'
Mog	5'-AGGCCTTGTATTCCTCTTCCTGC-3'
	5'-GCTCCAGGAAGACACAACCATCA-3'
Opalin	5'-AGCCCGTAGAGGAGACTGAGA-3'
	5'-TCTAGGCTCAGGCTGGGTACA-3'
Acsl3	5'-AGCCCGCGGGTCTTAC-3'
	5'-GATGGCAATCCACTCAGCAAC-3'
Dhcr24	5'-GTGAAGGGGTTGGAGTTCGT-3'
	5'-CTTCCATTCCCGGACCTGTT-3'
Hmgcs1	5'-CTATGATTGCATTGGGCGGC-3'
	5'-CCAGAGCATATCGTCCATCCC-3'
Sqle	5'-GCTGCTATTTTCCAGGCCAA-3'
	5'-CCAGAGGGTGAGGAGACAAT-3'
Lss	5'-TGCCTTTCTCTCTGGCCATG-3'
	5'-AGGGGAATGGGGATCAGACA-3'
Fa2h	5'-TCTGTCTCCTCTCTCCCTGC-3'
	5'-CCCTTCTTGGCTTCAGGAGG-3'

Transcript	Primers
Rpl8	5'-GTTCGTGTACTGCGGCAAGA-3'
	5'-ACAGGATTCATGGCCACACC-3'
Gapdh	5'-TCCAGTATGACTCTACCCACG-3'
	5'-CACGACATACTCAGCACCAG-3'

Supplementary table 3: Oligonucleotides for quantitative RT-PCR.

Locus	Primers
Wnt7a	5'-TGAACTGAGGCTTGCAAAAA-3'
	5'-AGCCTCTGAAAGTGGAGCAG-3'
Id4	5'-GATAAAGGAAGGCTTTCAGCAA-3'
	5'-TTGGATGGTGTGGTGCAG-3'
Tgfb2	5'-TTAGACTCCGGTTGGTGGAC-3'
	5'-AAACAATCATCGGCACAGAC-3'
Cspg4	5'-GACCATCTGTGAAAGAGACC-3'
	5'-GACAGAGGCGCGATTGTGCC-3'
Wnt7aneg	5'-GAAGATGTCTCATGGACAAC-3'
	5'-CAGTTTGGTATCAGGCATTC-3'
Tgfb2neg	5'-GAGTGTCTCATCATTTCATCT-3'
	5'-CAAGGTCTTGGATGCCTATG-3'
Cspg4neg	5'-CACTGGGATTAGATCTGAGCC-3'
	5'-CTGACCTGAAGAGATTCCTGC-3'

Supplementary table 4: Oligonucleotides for quantitative PCR in ChIP experiments.
